# Age and sex differences in oxytocin and vasopressin V1a receptor binding densities in the rat brain: focus on the social decision-making network

**DOI:** 10.1007/s00429-016-1260-7

**Published:** 2016-07-07

**Authors:** Caroline J. W. Smith, Max L. Poehlmann, Sara Li, Aarane M. Ratnaseelan, Remco Bredewold, Alexa H. Veenema

**Affiliations:** Neurobiology of Social Behavior Laboratory, Department of Psychology, Boston College, McGuinn 300, 140 Commonwealth Ave, Chestnut Hill, MA 02467 USA

**Keywords:** Oxytocin receptor, V1a receptor, Sex differences, Juvenile, Vasopressin, Oxytocin, Development

## Abstract

Oxytocin (OT) and vasopressin (AVP) regulate various social behaviors via activation of the OT receptor (OTR) and the AVP V1a receptor (V1aR) in the brain. Social behavior often differs across development and between the sexes, yet our understanding of age and sex differences in brain OTR and V1aR binding remains incomplete. Here, we provide an extensive analysis of OTR and V1aR binding density throughout the brain in juvenile and adult male and female rats, with a focus on regions within the social decision-making network. OTR and V1aR binding density were higher in juveniles than in adults in regions associated with reward and socio-spatial memory and higher in adults than in juveniles in key regions of the social decision-making network and in cortical regions. We discuss possible implications of these shifts in OTR and V1aR binding density for the age-specific regulation of social behavior. Furthermore, sex differences in OTR and V1aR binding density were less numerous than age differences. The direction of these sex differences was region-specific for OTR but consistently higher in females than in males for V1aR. Finally, almost all sex differences in OTR and V1aR binding density were already present in juveniles and occurred in regions with denser binding in adults compared to juveniles. Possible implications of these sex differences for the sex-specific regulation of behavior, as well potential underlying mechanisms, are discussed. Overall, these findings provide an important framework for testing age- and sex-specific roles of OTR and V1aR in the regulation of social behavior.

**Electronic supplementary material:**

The online version of this article (doi:10.1007/s00429-016-1260-7) contains supplementary material, which is available to authorized users.

## Introduction

Oxytocin (OT) and vasopressin (AVP) are neuropeptides primarily synthesized in the paraventricular and supraoptic nuclei of the hypothalamus. AVP is also synthesized in the bed nucleus of the stria terminalis (BNST) and medial amygdala (Sofroniew and Weindl 1978; Sofroniew 1980; Caffé et al. 1987; De Vries et al. 1981; De Vries and Buijs 1983; Rood and De Vries 2011). These OT and AVP-synthesizing nuclei send fiber projections to a wide array of brain regions (Buijs 1978, 1980; Knobloch and Grinevich 2014; Rood and De Vries 2011). Via these direct projections, as well as via dendritic release (Ludwig et al. 2002, 2005; Ludwig and Leng 2006), OT and AVP reach the OT receptor (OTR) and V1a receptor (V1aR), which are widely distributed in the brain (Gimpl and Fahrenholz 2001; Tribollet et al. 1990, 1998; Shapiro and Insel 1989; Dumais et al. 2013; Dumais and Veenema 2016a). By activating these receptors, OT and AVP have been shown to modulate a broad range of social behaviors in adult rodents, including social recognition, aggression, and maternal behavior (Veenema and Neumann 2008; Goodson and Kabelik 2009; Albers 2015).

In addition to regulating adult social behaviors, OT and AVP systems have been more recently implicated in the regulation of social behaviors during development. For example, OT and AVP systems were found to modulate a juvenile-typical and highly rewarding social behavior, namely social play behavior, in 35-day-old juvenile rats (Veenema et al. 2013; Bredewold et al. 2014). The juvenile period (here synonymous with the peri-pubertal or early adolescent period and spanning postnatal days 28–42 in rats (Spear 2000) is characterized by increased time spent engaging in peer interactions, novelty-seeking, and risk-taking behavior than at younger or older ages (Doremus-Fitzwater et al. 2010; Blakemore and Mills 2014). Juvenile animals also show more robust conditioned place preferences for peer interactions, suggesting that these interactions are more rewarding to juveniles than they are to adults (Douglas et al. 2004; Trezza et al. 2011; Crone and Dahl 2012). These findings suggest differences in the regulation of social behaviors between juveniles and adults. In support, the AVP system modulates social recognition differently in juvenile as compared to adult rats (Veenema et al. 2012). Such age differences in the regulation of social behavior by AVP, and possibly OT, may be due to age differences in OTR and V1aR expression in the brain.

Previous studies have characterized the developmental trajectory of OTR and V1aR binding densities in the rat brain from the prenatal period up through the peri-weaning period (as late as postnatal day 30) and compared these binding patterns with those in adult rats (Tribollet et al. 1989; Snijdewint et al. 1989; Shapiro and Insel 1989; Tribollet et al. 1991, 1992). However, to the best of our knowledge, only two studies so far have compared OTR and/or V1aR binding densities between 35-day-old juveniles and adults, albeit only in males and in a limited number of brain regions (Tribollet et al. 1989; Lukas et al. 2010). Furthermore, Tribollet et al. (1989) included only four animals in each group and did not provide quantitative statistical analysis of age differences between 35-day-old juveniles and adults. Thus, a comparison of differences in OTR and V1aR binding densities between juvenile and adult rats of both sexes, throughout the brain, is lacking.

OT and AVP often regulate social behaviors in sex-specific ways in adult rats (Bluthe and Dantzer 1990; Dantzer et al. 1987; Engelmann et al. 1998; Dumais et al. 2013; Lukas and Neumann 2014; Dumais and Veenema 2016a, b). This may be due to sex differences in OTR and V1aR expression in the brain. In support, sex differences have been found in OTR and V1aR binding densities in several regions of the adult rat brain with predominantly higher binding densities in males than in females (Dumais et al. 2013; Dumais and Veenema 2016a). Importantly, OT and AVP also regulate social behaviors in sex-specific ways in juvenile rats (Veenema et al. 2013; Bredewold et al. 2014), suggesting the presence of sex differences in OTR and V1aR binding densities prior to puberty. However, whether the sex differences in OTR and V1aR binding found in adult rats are already present in juvenile rats has yet to be determined.

We herein aim to provide a comprehensive comparison between juveniles and adults and between males and females of OTR and V1aR binding densities in the rat brain. We have particularly focused on analyzing OTR and V1aR binding densities in brain regions that are part of the social decision-making network (O’Connell and Hoffman 2011, 2012). This network combines brain regions of the mesolimbic reward system with those of the social behavior network (Newman 1999; O’Connell and Hoffman 2011, 2012) to form a reciprocally interconnected subset of brain regions involved in reward processing and behavioral regulation (O’Connell and Hoffman 2011, 2012). OTR and V1aR are expressed in most nodes of the social decision-making network (Albers 2015). Because there are age and sex differences in the reward value and expression of various social behaviors (Douglas et al. 2004; Varlinskaya et al. 2015; Panksepp et al. 1984; Terranova et al. 1993), we hypothesize that age and sex differences in OTR and V1aR binding densities will occur in multiple nodes of the social decision-making network. Furthermore, because sex differences in the regulation of social behavior by OT and AVP systems have been found in adults (Dantzer et al. 1987; Bluthe and Dantzer 1990; Veenema et al. 2012; Dumais et al. 2016), as well as in juveniles (Veenema et al. 2013; Bredewold et al. 2014), we hypothesize that sex differences in OTR and V1aR binding previously observed in adults (Dumais et al. 2013; Dumais and Veenema 2016a) will already be present in juveniles.

## Methods

### Animals

Male and female Wistar rats were obtained from Charles River Laboratories (Raleigh, NC, USA) at 22 or 56 days of age and housed under standard laboratory conditions (12 h light/dark cycle, lights on at 7:00 am, food and water available ad libitum, 22 °C, 60 % humidity). Upon arrival at our facility, rats were housed in standard rat cages (26.7 × 48.3 × 20.3 cm). Twenty-two-day-old juvenile rats were housed in same-sex groups of 3–4 until brain collection for receptor autoradiography at 35 days of age (Juvenile group). The age of 35 days was chosen to be consistent with previous work on OTR and V1aR binding in the brains of 35-day-old male rats (Lukas et al. 2010). Furthermore, 35 days of age marks a distinctive developmental stage in rats with peak levels of social play (Panksepp 1981; Pellis and Pellis 1990) a behavior modulated by activation of OTR and V1aR in the brain (Veenema et al. 2012, 2013; Bredewold et al. 2014). Fifty-six-day-old rats were housed in same-sex pairs until brain collection for receptor autoradiography at 84 days of age (Adult group). All experiments were conducted in accordance with the NIH *Guide to the Care and Use of Laboratory Animals* and approved by the Boston College Institutional Animal Care and Use Committee (IACUC).

### Receptor autoradiography

#### Coronal sectioning

**Fig. 1:**
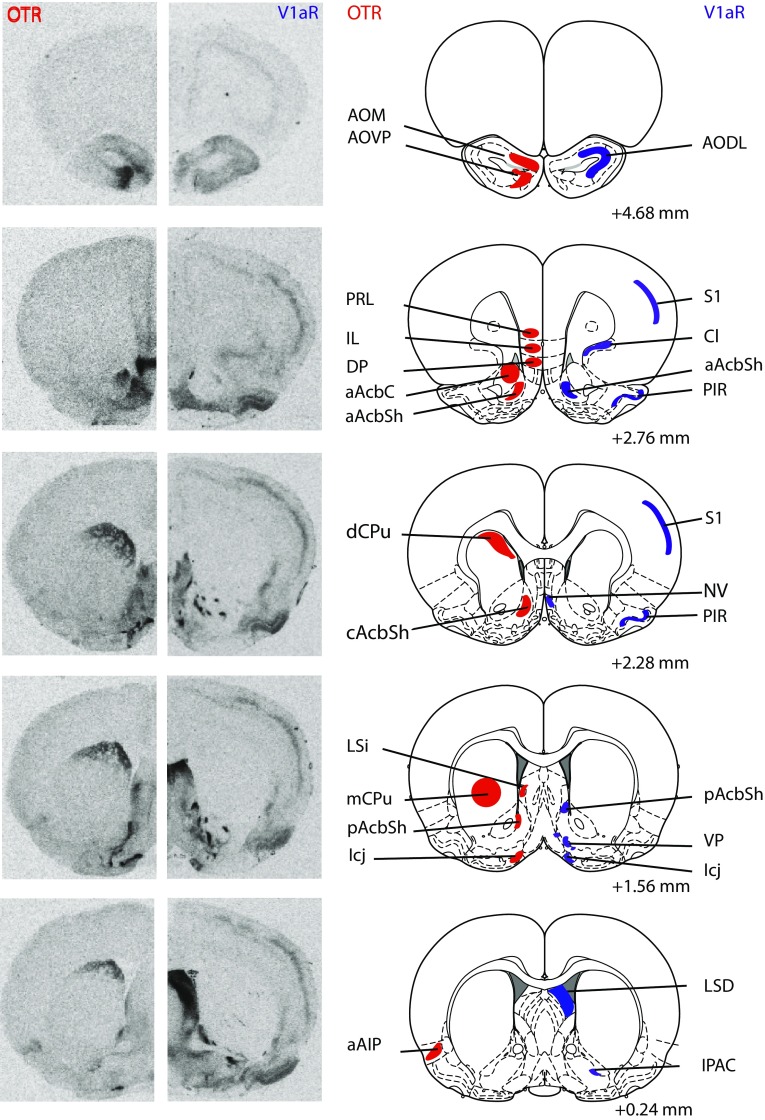

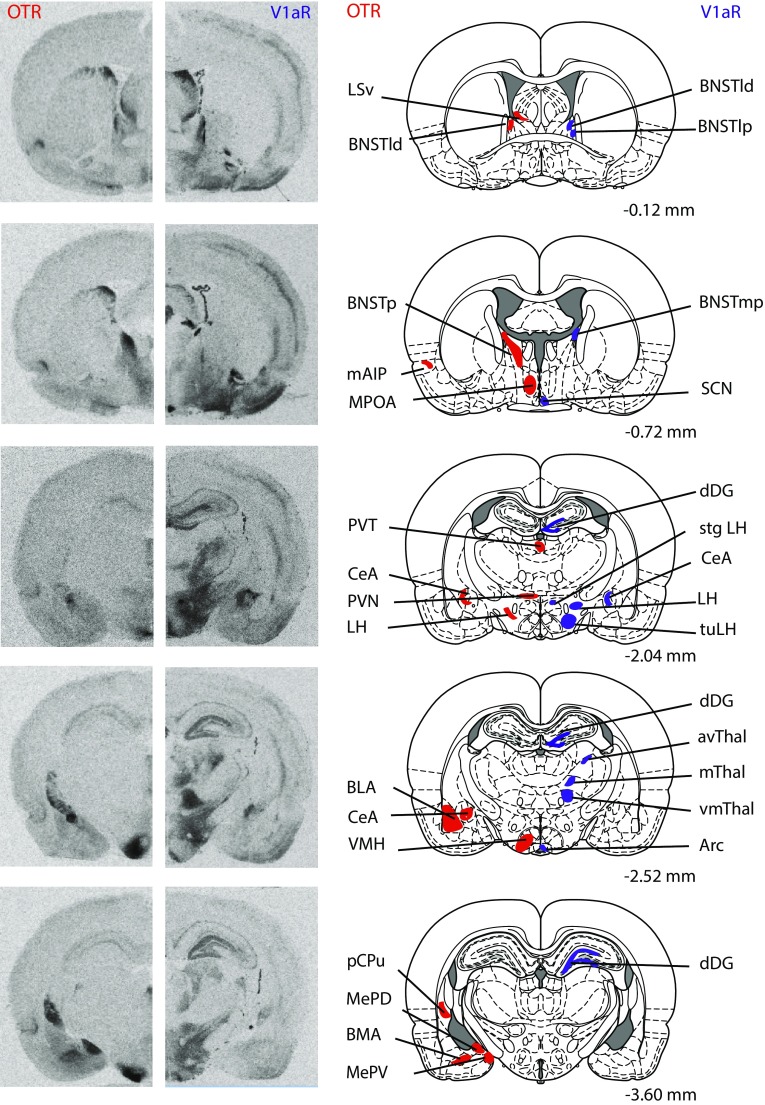

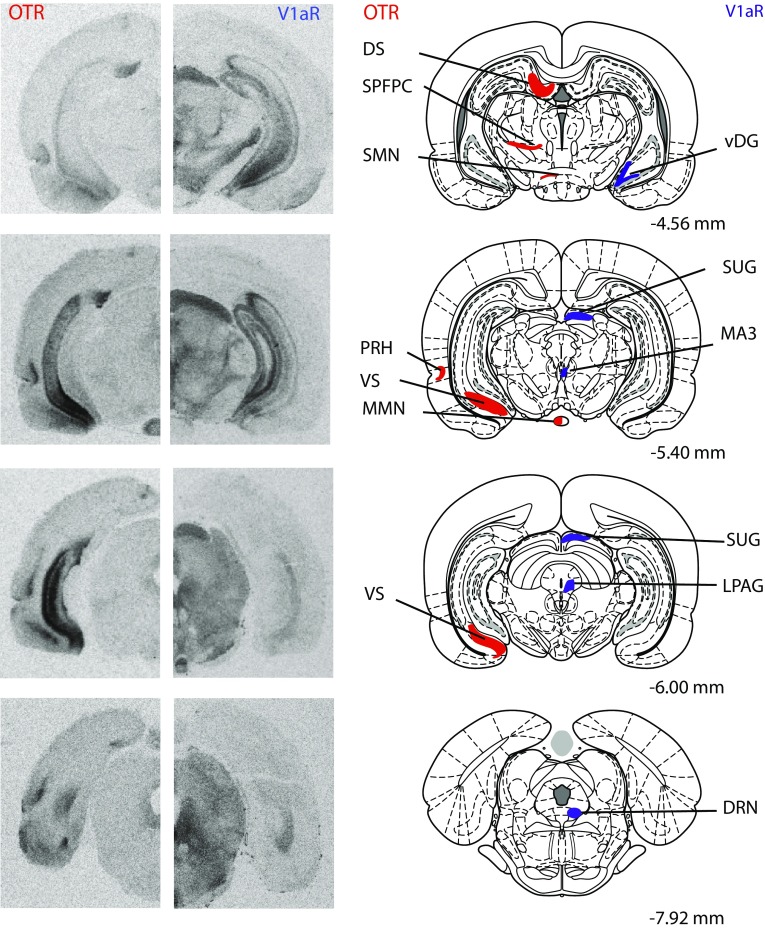
Representative autoradiograms of OTR and V1aR binding in coronal sections of the brain from the same adult male rat (on the *left*) with corresponding rat brain atlas images (Paxinos and Watson 2007; on the *right*). Brain regions in which receptor binding was measured are highlighted in *red* for OTR and are highlighted in *blue* for V1aR. Distance is measured in millimeters from bregma according to Paxinos and Watson (2007). Note that while many brain regions were analyzed across multiple bregma distances, regions are highlighted in the most representative atlas images only

Rats (juvenile males: *n* = 13; juvenile females: *n* = 13; adult males: *n* = 12; adult females: *n* = 12) were killed using CO_2_ inhalation and brains were removed, rapidly frozen in methylbutane on dry ice, and stored at −45 °C. Brains were cut on a cryostat into 16-µm coronal sections and mounted onto slides in eight adjacent series. Collection began at approximately 3.72 mm anterior to bregma and ended at approximately 8.52 mm posterior to bregma (Paxinos and Watson 2007). Sections were then frozen −45 °C until receptor autoradiography was performed. Receptor autoradiography was conducted for OTR using [^125^I]-Ornithine Vasotocin Analog (d(CH_2_)_5_[Tyr(Me)^2^,Thr^4^,Orn^8^,[^125^I]Tyr^9^-NH_2_]-OVTA; Perkin Elmer, Boston, MA, USA) as tracer and for V1aR using [^125^I]-d(CH_2_)_5_(Tyr[Me])-AVP (Perkin Elmer, Boston, MA, USA) as tracer on adjacent series. Specificity of these tracers to bind OTR and V1aR, respectively, has been demonstrated previously (Beery et al. 2008; Campbell et al. 2009; Anacker et al. 2016). Receptor autoradiography was conducted in accordance with Lukas et al. (2010). In brief, slides were thawed and dried at room temperature followed by a short fixation in 0.1 % paraformaldehyde. The slides were then washed twice in 50 mM Tris (pH 7.4), exposed to tracer buffer (50 pM tracer, 50 mM Tris, 10 mM MgCl2, 0.01 % BSA) for 60 min, and washed four times in Tris + 10 mM MgCl_2_. Finally, slides were dipped in distilled water, air-dried, and exposed to Biomax MR films (VWR International, Pittsburgh, PA, USA). Brain sections of both ages and sexes were processed together and balanced across incubation chambers and exposure to films. A 3-day exposure time was used to analyze OTR binding density in brain regions with relatively high OTR binding density (total of 13 regions); the medial anterior olfactory nucleus, ventroposterior anterior olfactory nucleus, anterior nucleus accumbens core, dorsal caudate putamen, medial caudate putamen, dorsal peduncular nucleus, islands of Calleja, posterior BNST, dorsolateral BNST, ventromedial hypothalamus, central amygdala, dorsal subiculum, and ventral subiculum. OTR binding density in additional regions with lower OTR binding density (21 regions) was analyzed using a 9-day exposure time. A 4-day exposure time was used to analyze V1aR binding density in a total of 29 brain regions. See Fig. 1 for receptor autoradiograms and schematic diagrams indicating the brain regions in which OTR and V1aR binding was quantified. All abbreviations of brain regions are in accordance with Paxinos and Watson (2007), except for the nucleus accumbens core and nucleus accumbens shell, where we added the subdivisions anterior core, medial shell, and posterior shell to delineate the separate areas analyzed as well as for the anterior olfactory nucleus where we used the abbreviation AODL to refer to measurements including both the dorsal and lateral divisions.

### Image and data analysis

Autoradiography films were digitized using a Northern Light Illuminator (InterFocus Imaging, UK) and optical densities of OTR and V1aR were measured in coronal sections using ImageJ (NIH, http://imagej.nih.gov/ij/). The data were converted to dpm/mg (disintegrations per minute/milligram tissue) using a [^125^I] standard microscale (American Radiolabeled Chemicals Inc., St. Louis, MO, USA). Each measurement was subtracted by film background and binding densities were calculated by taking the mean of bilateral measurements in a fixed number of sections per region of interest per rat. The total number of sections included depended on the size of the region of interest with a minimum of two sections. Regions of interest included those of the social decision-making network (see underlined brain regions in Table 1) as well as additional regions with dense OTR or V1aR binding. This resulted in 35 brain regions analyzed for OTR binding and 29 brain regions analyzed for V1aR binding. See Fig. 1 for receptor autoradiograms and schematic diagrams indicating all brain regions analyzed.

**Table 1:** Statistical details of age, sex, and interaction effects for OTR and V1aR binding densities in the rat brain

	OTR/V1aR	Direction	Age effect	Sex effect	Interaction effect
Cortical areas					
aAIP	OTR	Higher in adults and females	***F***_**(1,41)**_ **=** **48.2;** ***p*** **<** **0.001**	***F***_**(1,41)**_ **=** **7.80;** ***p*** **<** **0.005**	*F*_(1,41)_ **=** 0.64; *p* **=** 0.43
mAIP	OTR	Higher in adults	***F***_**(1,45)**_ **=** **21.0;** ***p*** **<** **0.001**	*F*_(1,45)_ **=** 4.14; *p* **=** 0.05	*F*_(1,45)_ **=** 0.03; *p* **=** 0.86
DP	OTR		*F*_(1,46)_ **=** 0.97; *p* **=** 0.33	*F*_(1,46)_ **=** 0.67; *p* **=** 0.42	*F*_(1,46)_ **=** 2.60; *p* **=** 0.11
Il	OTR	Higher in adults	***F***_**(1,46)**_ **=** **7.54;** ***p*** **<** **0.001**	*F*_(1,46)_ **=** 0.01; *p* **=** 0.99	*F*_(1,46)_ **=** 0.01; *p* **=** 0.94
Pir	V1aR	Higher in adults	***F***_**(1,44)**_ **=** **11.7;** ***p*** **<** **0.001**	*F*_(1,44)_ **=** 0.10; *p* **=** 0.75	*F*_(1,44)_ **=** 0.02; *p* **=** 0.90
PRh	OTR	Higher in adults and females	***F***_**(1,44)**_ **=** **15.2;** ***p*** **<** **0.001**	***F***_**(1,44)**_ **=** **7.80;** ***p*** **<** **0.001**	*F*_(1,44)_ **=** 1.41; *p* **=** 0.24
PRL	OTR	Higher in adults	***F***_**(1,46)**_ **=** **18.8;** ***p*** **<** **0.001**	*F*_(1,46)_ **=** 3.14; *p* **=** 0.08	*F*_(1,46)_ **=** 0.01; *p* **=** 0.99
S1	V1aR	Higher in adults	***F***_**(1,44)**_ **=** **23.5;** ***p*** **<** **0.001**	*F*_(1,44)_ **=** 2.65; *p* **=** 0.11	*F*_(1,44)_ **=** 0.26; *p* **=** 0.62
Olfactory areas					
AOM	OTR	Higher in juveniles	***F***_**(1,23)**_ **=** **16.4;** ***p*** **<** **0.001**	*F*_(1,23)_ **=** 0.88; *p* **=** 0.36	*F*_(1,23)_ **=** 0.71; *p* **=** 0.41
AOPV	OTR	Higher in juveniles	***F***_**(1,22)**_ **=** **29.6;** ***p*** **<** **0.001**	*F*_(1,22)_ **=** 3.54; *p* **=** 0.07	*F*_(1,22)_ **=** 0.15; *p* **=** 0.71
AODL	V1aR	Higher in juveniles	***F***_**(1,20)**_ **=** **8.59;** ***p*** **<** **0.01**	*F*_(1,20)_ **=** 0.06; *p* **=** 0.80	*F*_(1,20)_ **=** 0.33; *p* **=** 0.57
Nv	V1aR	Higher in adults	***F***_**(1,44)**_ **=** **12.5;** ***p*** **<** **0.001**	*F*_(1,44)_ **=** 0.49; *p* **=** 0.49	*F*_(1,44)_ **=** 3.26; *p* **=** 0.08
Striatal areas					
aAcbC	OTR	Higher in juveniles	***F***_**(1,46)**_ **=** **84.3;** ***p*** **<** **0.001**	*F*_(1,46)_ **=** 0.02; *p* **=** 0.88	*F*_(1,46)_ **=** 1.84; *p* **=** 0.18
aAcbSh	OTR		*F*_(1,44)_ **=** 4.02; *p* **=** 0.051	*F*_(1,44)_ **=** 0.31; *p* **=** 0.58	*F*_(1,44)_ **=** 0.13; *p* **=** 0.72
aAcbSh	V1aR		*F*_(1,39)_ **=** 0.01; *p* **=** 0.97	*F*_(1,39)_ **=** 1.03; *p* **=** 0.32	*F*_(1,39)_ **=** 4.35; *p* **=** 0.04
cAcbSh	OTR		*F*_(1,45)_ **=** 1.18; *p* **=** **0.28**	*F*_(1,45)_ **=** 0.41; *p* **=** 0.53	*F*_(1,45)_ **=** 0.44; *p* **=** 0.51
pAcbSh	OTR		*F*_(1,46)_ **=** 0.77; *p* **=** 0.39	*F*_(1,46)_ **=** 0.01; *p* **=** 0.99	*F*_(1,46)_ **=** 0.05; *p* **=** 0.83
pAcbSh	V1aR	Higher in adults	***F***_**(1,44)**_ **=** **47.4;** ***p*** **<** **0.001**	*F*_(1,44)_ **=** 1.35; *p* **=** 0.25	*F*_(1,44)_ **=** 0.64; *p* **=** 0.43
dCPu	OTR	Higher in juveniles	***F***_**(1,40)**_ **=** **80.0;** ***p*** **<** **0.001**	*F*_(1,40)_ **=** 1.89; *p* **=** 0.18	*F*_(1,40)_ **=** 0.32; *p* **=** 0.57
mCPu	OTR	Higher in juveniles	***F***_**(1,46)**_ **=** **6.35;** ***p*** **=** **0.015**	*F*_(1,46)_ **=** 0.02; *p* **=** 0.88	*F*_(1,46)_ **=** 0.04; *p* **=** 0.84
pCPu	OTR	Higher in adult females	*F*_(1,46)_ **=** 3.54; *p* **=** 0.07	*F*_(1,46)_ **=** 0.26; *p* **=** 0.61	***F***_**(1,46)**_ **=** **6.34,** ***p*** **=** **0.015**
ICj	OTR	Higher in adult males	***F***_**(1,45)**_ **=** **175;** ***p*** **<** **0.001**	***F***_**(1,45)**_ **=** **80.2;** ***p*** **<** **0.001**	***F***_**(1,45)**_ **=** **99.6,** ***p*** **<** **0.001**
ICj	V1aR	Higher in juveniles	***F***_**(1,44)**_ **=** **11.3;** ***p*** **<** **0.005**	*F*_(1,44)_ **=** 1.79; *p* **=** 0.19	*F*_(1,44)_ **=** 0.42; *p* **=** 0.52
VP	V1aR	Higher in adults	***F***_**(1,44)**_ **=** **26.7;** ***p*** **<** **0.001**	*F*_(1,44)_ **=** 1.72; *p* **=** 0.20	*F*_(1,44)_ **=** 0.42; *p* **=** 0.52
Lateral septum					
LSD	V1aR	Higher in adults and females	***F***_**(1,42)**_ **=** **22.8;** ***p*** **<** **0.001**	***F***_**(1,42)**_ **=** **9.14;** ***p*** **<** **0.005**	*F*_(1,42)_ **=** 0.92; *p* **=** 0.34
LSI	OTR	Higher in females	*F*_(1,46)_ **=** 4.0; *p* **=** 0.50	***F***_**(1,46)**_ **=** **8.80;** ***p*** **<** **0.01**	*F*_(1,46)_ **=** 1.34; *p* **=** 0.25
LSV	OTR	Higher in juveniles	***F***_**(1,46)**_ **=** **34.3;** ***p*** **<** **0.001**	*F*_(1,46)_ **=** 0.02; *p* **=** 0.88	*F*_(1,46)_ **=** 0.53; *p* **=** 0.47
BNST					
BNSTld	OTR		*F*_(1,45)_ **=** 2.53; *p* **=** 0.12	*F*_(1,45)_ **=** 1.62; *p* **=** 0.21	*F*_(1,45)_ **=** 1.22; *p* **=** 0.27
BNSTld	V1aR		*F*_(1,43)_ **=** 3.83; *p* **=** 0.06	*F*_(1,43)_ **=** 1.91; *p* **=** 0.17	*F*_(1,43)_ **=** 0.04; *p* **=** 0.85
BNSTlp	V1aR		*F*_(1,43)_ **=** 5.25; *p* **=** 0.03	*F*_(1,43)_ **=** 5.42; *p* **=** 0.03	*F*_(1,43)_ **=** 0.16; *p* **=** 0.69
BNSTmp	V1aR		*F*_(1,43)_ **=** 0.55; *p* **=** 0.47	*F*_(1,43)_ **=** 0.01; *p* **=** 0.98	*F*_(1,43)_ **=** 0.16; *p* **=** 0.69
BNSTp	OTR	Higher in adults and males	***F***_**(1,41)**_ **=** **6.89;** ***p*** **<** **0.05**	***F***_**(1,41)**_ **=** **136;** ***p*** **<** **0.001**	*F*_(1,41)_ **=** 4.07; *p* **=** 0.05
Amygdala					
BLA	OTR	Higher in juveniles	***F***_**(1,45)**_ **=** **7.99;** ***p*** **<** **0.005**	*F*_(1,45)_ **=** 3.50; *p* **=** 0.07	*F*_(1,45)_ **=** 2.61; *p* **=** 0.11
BMA	OTR	Higher in juveniles	***F***_**(1,45)**_ **=** **8.96;** ***p*** **<** **0.005**	*F*_(1,45)_ **=** 0.03; *p* **=** 0.86	*F*_(1,45)_ **=** 5.54; *p* **=** 0.02
CeA	OTR		*F*_(1,46)_ **=** 0.01; *p* **=** 0.96	*F*_(1,46)_ **=** 1.84; *p* **=** 0.18	*F*_(1,46)_ **=** 2.38; *p* **=** 0.13
CeA	V1aR	Higher in juveniles	***F***_**(1,44)**_ **=** **10.3;** ***p*** **<** **0.005**	*F*_(1,44)_ **=** 1.99; *p* **=** 0.17	*F*_(1,44)_ **=** 0.40; *p* **=** 0.53
Cl	V1aR	Higher in juveniles	***F***_**(1,44)**_ **=** **26.1;** ***p*** **<** **0.001**	*F*_(1,44)_ **=** 0.49; *p* **=** 0.49	*F*_(1,44)_ **=** 0.09; *p* **=** 0.77
IPAC	V1aR	Higher in adults	***F***_**(1,44)**_ **=** **7.08;** ***p*** **=** **0.011**	*F*_(1,44)_ **=** 0.04; *p* **=** 0.85	*F*_(1,44)_ **=** 0.46; *p* **=** 0.50
MePD	OTR	Higher in adult males	***F***_**(1,46)**_ **=** **23.0;** ***p*** **<** **0.001**	***F***_**(1,46)**_ **=** **252;** ***p*** **<** **0.001**	***F***_**(1,46)**_ **=** **26.4,** ***p*** **<** **0.001**
MePV	OTR	Higher in males	*F*_(1,46)_ **=** 0.34; *p* **=** 0.56	***F***_**(1,46)**_ **=** **35.1;** ***p*** **<** **0.001**	*F*_(1,46)_ **=** 0.42; *p* **=** 0.52
Hypothalamus					
Arc	V1aR	Higher in adults and females	***F***_**(1,44)**_ **=** **50.5;** ***p*** **<** **0.001**	***F***_**(1,44)**_ **=** **30.6;** ***p*** **<** **0.001**	*F*_(1,44)_ **=** 0.29; *p* **=** 0.60
LH	OTR		*F*_(1,41)_ **=** 0.35; *p* **=** 0.56	*F*_(1,41)_ **=** 0.29; *p* **=** 0.59	*F*_(1,41)_ **=** 0.01; *p* **=** 0.99
LH	V1aR		*F*_(1,42)_ **=** 0.13; *p* **=** 0.72	*F*_(1,42)_ **=** 0.30; *p* **=** 0.58	*F*_(1,42)_ **=** 0.06; *p* **=** 0.81
MMN	OTR	Higher in juveniles	***F***_**(1,40)**_ **=** **36.0;** ***p*** **<** **0.001**	*F*_(1,40)_ **=** 3.50; *p* **=** 0.07	*F*_(1,40)_ **=** 2.82; *p* **=** 0.10
MPOA	OTR	Higher in adults	***F***_**(1,43)**_ **=** **50.0;** ***p*** **<** **0.001**	*F*_(1,43)_ **=** 4.32; *p* **=** 0.04	*F*_(1,43)_ **=** 0.57; *p* **=** 0.46
PVN	OTR	Higher in juveniles and males	***F***_**(1,41)**_ **=** **18.2;** ***p*** **<** **0.001**	***F***_**(1,41)**_ **=** **11.5;** ***p*** **<** **0.005**	*F*_(1,41)_ **=** 0.01; *p* **=** 0.91
SCN	V1aR		*F*_(1,44)_ **=** 1.06; *p* **=** 0.31	*F*_(1,44)_ **=** 2.31; *p* **=** 0.14	*F*_(1,44)_ **=** 1.71; *p* **=** 0.20
SMN	OTR	Higher in juveniles	***F***_**(1,45)**_ **=** **20.6;** ***p*** **<** **0.001**	*F*_(1,45)_ **=** ** 0.86**; *p* **=** 0.36	*F*_(1,45)_ **=** 4.61; *p* **=** 0.04
Stg	V1aR	Higher in juveniles	***F***_**(1,44)**_ **=** **14.0;** ***p*** **<** **0.005**	*F*_(1,44)_ **=** 0.38; *p* **=** 0.54	*F*_(1,44)_ **=** 0.06; *p* **=** 0.81
tuLH	V1aR		*F*_(1,42)_ **=** 0.06; *p* **=** 0.82	*F*_(1,42)_ **=** 2.41; *p* **=** 0.13	*F*_(1,42)_ **=** 1.70; *p* **=** 0.20
VMH	OTR	Higher in adult males	***F***_**(1,46)**_ **=** **269;** ***p*** **<** **0.001**	***F***_**(1,46)**_ **=** **15.5;** ***p*** **<** **0.001**	***F***_**(1,46)**_ **=** **12.2,** ***p*** **<** **0.001**
Thalamus					
avThal	V1aR		*F*_(1,44)_ **=** 0.66; *p* **=** 0.42	*F*_(1,44)_ **=** 5.57; *p* **=** 0.02	*F*_(1,44)_ **=** 1.43; *p* **=** 0.24
mThal	V1aR		*F*_(1,44)_ **=** 2.40; *p* **=** 0.13	*F*_(1,44)_ **=** 0.06; *p* **=** 0.80	*F*_(1,44)_ **=** 0.14; *p* **=** 0.71
PVT	OTR	Higher in juveniles	***F***_**(1,41)**_ **=** **40.1;** ***p*** **<** **0.001**	*F*_(1,41)_ **=** 3.34; *p* **=** 0.08	*F*_(1,41)_ **=** 0.04; *p* **=** 0.85
SPFPC	OTR	Higher in juveniles	***F***_**(1,39)**_ **=** **13.5;** ***p*** **<** **0.005**	*F*_(1,39)_ **=** 0.07; *p* **=** 0.79	*F*_(1,39)_ **=** 0.77; *p* **=** 0.39
vmThal	V1aR	Higher in adults and females	***F***_**(1,44)**_ **=** **130;** ***p*** **<** **0.001**	***F***_**(1,44)**_ **=** **29.3;** ***p*** **<** **0.001**	*F*_(1,44)_ **=** 4.95; *p* **=** 0.03
Hippocampus					
DS	OTR	Higher in juveniles	***F***_**(1,46)**_ **=** **89.0;** ***p*** **<** **0.001**	*F*_(1,46)_ **=** 0.90; *p* **=** 0.35	*F*_(1,46)_ **=** 0.19; *p* **=** 0.67
VS	OTR	Higher in juveniles	***F***_**(1,43)**_ **=** **37.1;** ***p*** **<** **0.001**	*F*_(1,43)_ **=** 1.88; *p* **=** 0.18	*F*_(1,43)_ **=** 3.69; *p* **=** 0.06
dDG	V1aR	Higher in juveniles	***F***_**(1,44)**_ **=** **79.9;** ***p*** **<** **0.001**	*F*_(1,44)_ **=** 0.26; *p* **=** 0.61	*F*_(1,44)_ **=** 0.30; *p* **=** 0.59
vDG	V1aR	Higher in juveniles	***F***_**(1,38)**_ **=** **16.3;** ***p*** **<** **0.001**	*F*_(1,38)_ **=** 0.56; *p* **=** 0.46	*F*_(1,38)_ **=** 0.05; *p* **=** 0.83
Midbrain					
DRN	V1aR		*F*_(1,36)_ **=** 2.66; *p* **=** 0.11	*F*_(1,36)_ **=** 5.77; *p* **=** 0.02	*F*_(1,36)_ **=** 0.55; *p* **=** 0.46
LPAG	V1aR		*F*_(1,44)_ **=** 3.75; *p* **=** 0.06	*F*_(1,44)_ **=** 2.36; *p* **=** 0.13	*F*_(1,44)_ **=** 2.06; *p* **=** 0.16
MA3	V1aR	Higher in juveniles	***F***_**(1,44)**_ **=** **9.75;** ***p*** **<** **0.005**	*F*_(1,44)_ **=** 0.01; *p* **=** 0.94	*F*_(1,44)_ **=** 1.04; *p* **=** 0.31
Sug	V1aR		*F*_(1,44)_ **=** 4.79; *p* **=** 0.03	*F*_(1,44)_ **=** 0.01; *p* **=** 0.91	*F*_(1,44)_ **=** **3.97**; *p* **=** 0.05

Significant effects (two-way ANOVA with FDR correction: *α* < 0.020 for OTR and *α* < 0.015 for V1aR) are bolded. Underlined brain regions are part of the social decision-making network according to O’Connell and Hoffman (2011, 2012)

### Statistics

For all statistical analysis, PASW/SPSS Statistics (Version 22.0) was used. Two-way ANOVAs were used to test for age and sex differences in OTR and V1aR binding density in each brain region. The false discovery rate (FDR) procedure was used to correct for multiple comparisons (age, sex, and interaction) for each receptor separately. This resulted in an FDR *α* < 0.020 for OTR (based on 105 comparisons) and an FDR *α* < 0.015 for V1aR (based on 87 comparisons) (Hochberg and Benjamini 1990). Significant interaction effects were followed by Bonferroni post hoc tests (reflecting *t* tests pre-adjusted for multiple comparisons) to examine differences among groups. Significant age or sex effects were followed by Cohen’s *d* to calculate the effect size of age differences (overall and separately for male and females) and of sex differences (overall and separately for juveniles and adults). Subsequent independent samples *t* tests were run separately for OTR and V1aR to determine whether the effect size of age differences was different between males and females for all brain regions and whether the effect size of sex differences was different between juveniles and adults. Bivariate correlation analyses were used to determine correlations of OTR and/or V1aR binding densities between pre-selected brain regions based on patterns of age and/or sex differences in OTR and V1aR binding densities. Given the exploratory nature of our correlations we did not include a correction for multiple comparisons. Significance for correlation analyses was set at *p* < 0.05.

## Results

### Age differences in OTR binding density

**Fig. 2:**
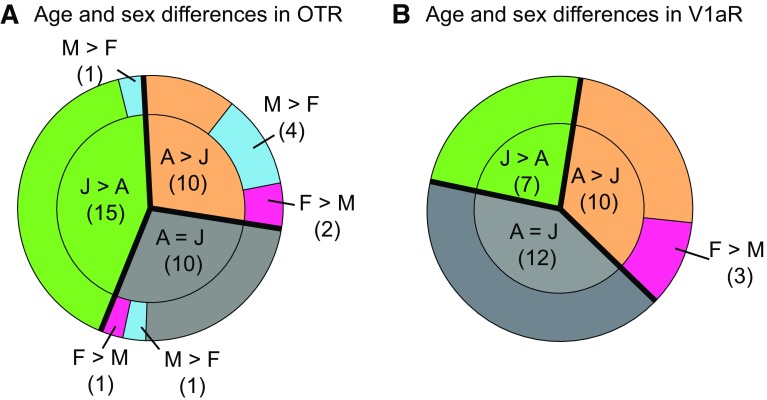
Overview of age and sex differences in OTR and V1aR binding densities in the rat brain. Age differences (*green* and *orange*) are more prevalent than sex differences (*blue* and *pink*) in both OTR binding density (**a**) and V1aR binding density (**b**). Data represent proportions of brain regions that significantly differ by age and sex for OTR or V1aR determined by two-way ANOVA with FDR correction for multiple comparisons. The number of brain regions is indicated in *parentheses*. *J* juveniles, *A* adults, *M* males, *F* females

**Fig. 3:**
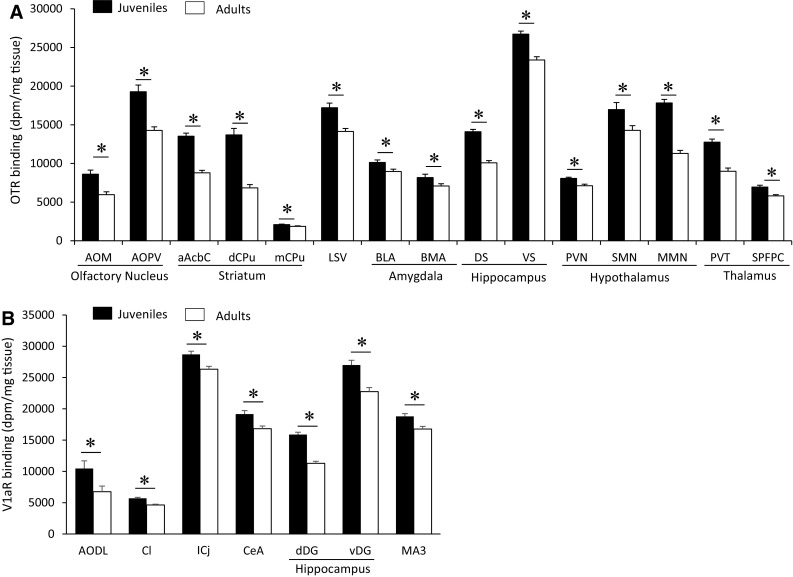
Brain regions in which OTR **a** and V1aR, **b** binding density is higher in juveniles than in adults. OTR binding was analyzed on 3-day exposure films for subregions of the olfactory nucleus, striatum, and hippocampus and on 9-day exposure films for all other regions. V1aR binding was analyzed on 4-day exposure films. *Bars* indicate mean + SEM; two-way ANOVA (age × sex) with FDR correction for multiple comparisons; data are collapsed across sexes to highlight main effects of age: *FDR *α* < 0.020 (**a**) and *FDR *α* < 0.015 (**b**)

**Fig. 4:**
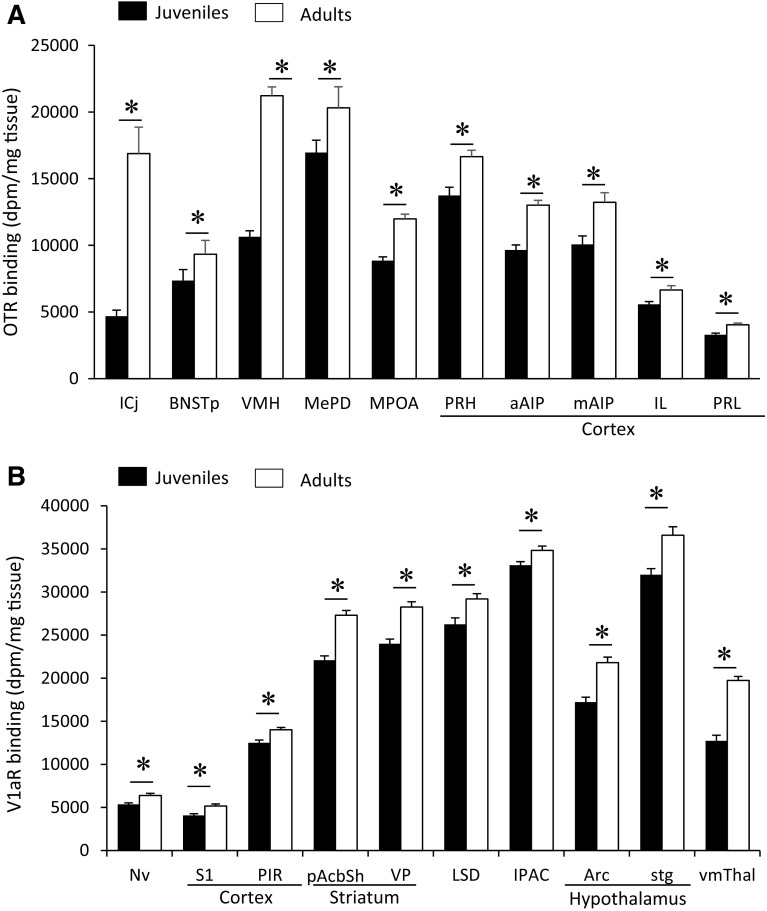
Brain regions in which OTR **a** and V1aR **b** binding density is higher in adults than in juveniles. OTR binding was analyzed on 3-day exposure films for the ICj, BNSTp and VMH and on 9-day exposure films for all other regions. V1aR binding was analyzed on 4-day exposure films. *Bars* indicate mean + SEM; two-way ANOVA (age × sex) with FDR correction for multiple comparisons; data are collapsed across sexes to highlight main effects of age: *FDR *α* < 0.020 (**a**) and *FDR *α* < 0.015 (**b**)

**Fig. 5:**
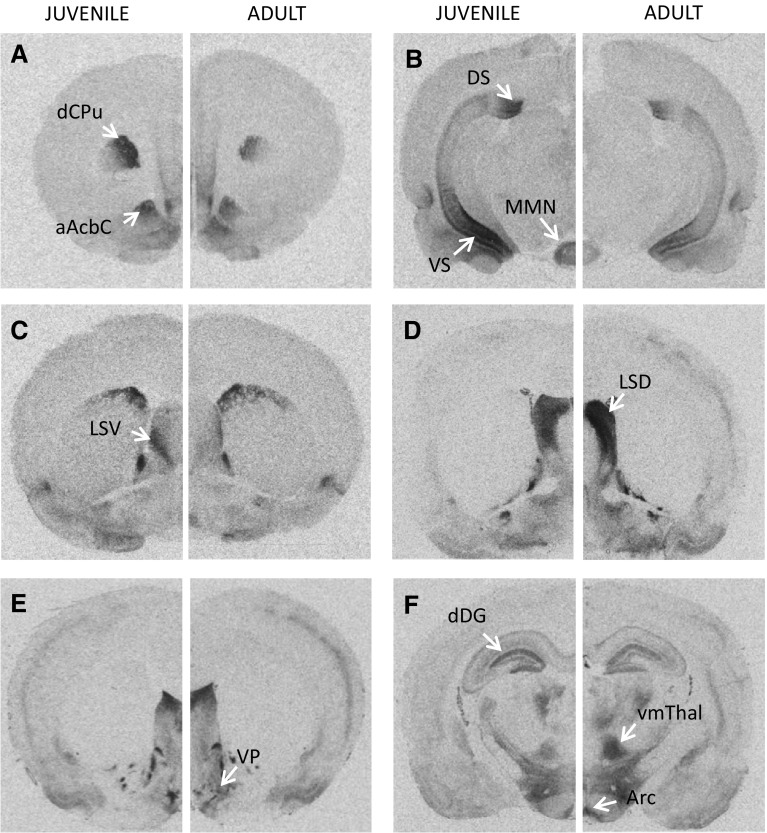
Representative images of age differences in OTR (**a**–**c**) and V1aR (**d**–**f**) binding densities. OTR binding density is higher in juveniles than adults in the dCPu and aAcbC (**a**), in the DS, VS, and MMN (**b**), and in the LSV (**c**). V1aR binding density is higher in adults than juveniles in the LSD (**d**), VP (**e**), Arc, and vmThal (**f**), but higher in juveniles than adults in the dDG (**f**). Images represent autoradiograms of a juvenile male and an adult male

Age differences in OTR binding density were found in 25 of the 35 brain regions analyzed (Fig. 2a; see Table 1 for complete statistics and Fig. 5 for representative images). OTR binding density was significantly higher in juveniles than in adults in 15 brain regions, consisting of subregions in the olfactory nucleus (medial and posteroventral anterior), striatum (anterior nucleus accumbens core, dorsal caudate putamen, medial caudate putamen), hypothalamus (paraventricular, medial mammillary, and supramammillary nuclei), amygdala (basolateral and basomedial), septum (ventral lateral), hippocampus (dorsal and ventral subiculum), and thalamus (paraventricular nucleus, subparafascicular nucleus) (Fig. 3a). OTR binding density was higher in adults as compared to juveniles in 10 brain regions: the islands of Calleja, ventromedial hypothalamus, posterior BNST, posterodorsal medial amygdala, medial preoptic area, and prelimbic, infralimbic, anterior and medial insular, and perirhinal cortices (Fig. 4a). While the size of individual age differences sometimes differed between males and females (see Table 2 for details), we found no overall difference in the effect sizes of age differences in OTR binding between the sexes (*t*_(48)_ **=** −0.51; *p* = 0.61).

**Table 2:** Cohen’s *d* effect size measurements for age differences in OTR and V1aR binding densities overall, and analyzed separately in males and females

	OTR/V1aR	Direction	Both sexes	Males	Females
Cortical areas					
aAIP	OTR	Higher in adults	−1.92	−2.64	−1.89
mAIP	OTR	Higher in adults	−1.29	−1.66	−1.09
Il	OTR	Higher in adults	−0.79	−0.72	−0.82
Pir	V1aR	Higher in adults	−1.03	−0.93	−1.15
PRh	OTR	Higher in adults	−1.08	−1.49	−0.78
PRL	OTR	Higher in adults	−1.21	−1.05	−1.56
S1	V1aR	Higher in adults	−1.35	−1.38	−1.40
Olfactory areas					
AOM	OTR	Higher in juveniles	1.56	1.90	1.26
AOPV	OTR	Higher in juveniles	2.01	2.07	2.30
AODL	V1aR	Higher in juveniles	1.19	1.00	1.37
NV	V1aR	Higher in adults	−1.03	−1.32	−0.65
Striatal areas					
aAcbC	OTR	Higher in juveniles	2.62	2.29	2.92
pAcbSh	V1aR	Higher in adults	−2.00	−1.52	−2.79
dCPu	OTR	Higher in juveniles	2.84	2.71	3.10
mCPu	OTR	Higher in juveniles	0.73	0.69	0.73
pCPu	OTR	Higher in adult females	−0.51	0.20	−1.17
Icj	OTR	Higher in adult males	−1.73	−6.79	−0.90
Icj	V1aR	Higher in juveniles	1.01	1.35	0.71
VP	V1aR	Higher in adults	−1.50	−1.50	−1.49
Lateral septum					
LSD	V1aR	Higher in adults	−1.25	−1.46	−1.54
LSV	OTR	Higher in juveniles	1.70	2.62	1.20
BNST					
BNSTp	OTR	Higher in adults	−0.44	−1.28	−0.21
Amygdala					
BLA	OTR	Higher in juveniles	0.76	1.19	0.38
BMA	OTR	Higher in juveniles	0.82	1.34	0.23
CeA	V1aR	Higher in juveniles	0.95	1.07	0.91
Cl	V1aR	Higher in juveniles	1.52	1.54	1.48
IPAC	V1aR	Higher in adults	−0.78	−0.69	−0.83
MePD	OTR	Higher in adults	−0.52	−2.81	0.10
Hypothalamus					
Arc	V1aR	Higher in adults	−1.56	−2.31	−1.8
MMN	OTR	Higher in juveniles	1.76	2.95	1.17
MPOA	OTR	Higher in adults	−1.99	−2.16	−2.04
PVN	OTR	Higher in juveniles	1.13	1.13	1.44
SMN	OTR	Higher in juveniles	1.27	1.78	0.76
Stg	V1aR	Higher in adults	−1.13	−1.17	−1.04
VMH	OTR	Higher in adults	−3.75	−5.25	−3.99
Thalamus					
PVT	OTR	Higher in juveniles	1.93	1.82	2.07
SPFPC	OTR	Higher in juveniles	1.20	1.38	0.92
vmThal	V1aR	Higher in adults	−2.51	−3.99	−2.73
Hippocampus					
DS	OTR	Higher in juveniles	2.70	2.87	2.49
VS	OTR	Higher in juveniles	1.72	1.25	2.31
dDG	V1aR	Higher in juveniles	2.66	2.41	2.88
vDG	V1aR	Higher in juveniles	1.30	1.44	1.14
Midbrain					
MA3	V1aR	Higher in juveniles	0.94	1.14	0.66

Only brain regions showing significant main effects of age or interaction (see Table 1) are included

### Age differences in V1aR binding density

Age differences in V1aR binding density were found in 17 of the 29 brain regions analyzed (Fig. 2b; see Table 1 for complete statistics and Fig. 5 for representative images). V1aR binding density was higher in juveniles as compared to adults in seven brain regions: the dorsolateral anterior olfactory nucleus, anterior claustrum, islands of Calleja, central amygdala, dentate gyrus (granular layer and molecular layer), and oculomotor nucleus (Fig. 3b). Adults had higher V1aR binding density than juveniles in 10 brain regions: the primary somatosensory and piriform cortices, posterior nucleus accumbens shell, ventral pallidum, arcuate nucleus, navicular nucleus, dorsal lateral septum, stigmoid hypothalamic nucleus, ventromedial thalamic nucleus, and interstitial nucleus of the posterior limb of the anterior commissure (Fig. 4b). While the size of individual age differences sometimes differed between males and females (see Table 2 for details), we found no overall difference in the effect sizes of age differences in V1aR binding between the sexes (*t*_(32)_ = −0.007; *p* = 0.99).

### Sex differences in OTR binding density

**Fig. 6:**
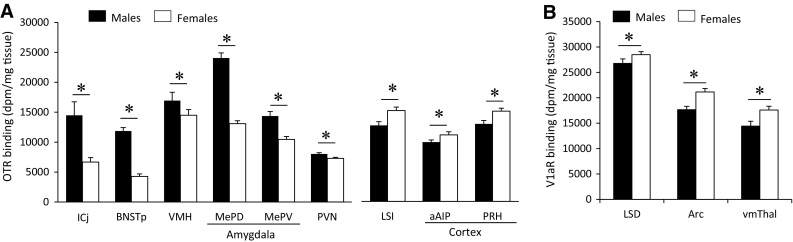
Brain regions in which OTR **a** and V1aR **b** binding densities show sex differences. OTR binding was analyzed on 3-day exposure films for the ICj, BNSTp and VMH and on 9-day exposure films for all other regions. V1aR binding was analyzed on 4-day exposure films. *Bars* indicate mean + SEM; two-way ANOVA (age × sex) with FDR correction for multiple comparisons; data collapsed across ages to highlight main effects of sex: *FDR *α* < 0.020 (**a**) and *FDR *α* < 0.015 (**b**)

Sex differences in OTR binding density were found in 9 of the 35 brain regions analyzed (Fig. 2a; see Table 1 for complete statistics). Males had higher OTR binding density than females in six brain regions: the islands of Calleja, posterior BNST, ventromedial hypothalamus, posterior-dorsal and posterior-ventral medial amygdala, and paraventricular nucleus of the hypothalamus (Fig. 6a). Females had higher OTR binding than males in three brain regions: the anterior insular cortex, perirhinal cortex, and intermediate lateral septum (Fig. 6a). While the size of individual sex differences sometimes differed between juveniles and adults (see Table 3 for details), we found no overall difference in the effect sizes of sex differences in OTR binding between ages (*t*_(18)_ = −1.35; *p* = 0.19).

**Table 3:** Cohen’s *d* effect size measurements for sex differences in OTR and V1aR binding densities overall, and analyzed separately in juveniles and adults

	OTR/V1aR	Direction	Both sexes	Juveniles	Adults
Cortical areas					
aAIP	OTR	Higher in females	−0.56	−0.58	−1.11
PRh	OTR	Higher in females	−0.82	−1.15	−0.59
Striatal areas					
pCPu	OTR	Higher in adult females	−0.11	−0.49	−1.10
Icj	OTR	Higher in adult males	0.93	−0.41	4.38
Lateral septum					
LSD	V1aR	Higher in females	−0.65	−1.14	−0.68
LSI	OTR	Higher in females	−0.82	−1.25	−0.48
BNST					
BNSTp	OTR	Higher in males	3.19	2.57	4.93
Amygdala					
MePD	OTR	Higher in males	3.15	2.64	7.39
MePV	OTR	Higher in males	1.69	1.36	2.10
Hypothalamus					
Arc	V1aR	Higher in females	−1.06	−1.93	−1.32
PVN	OTR	Higher in males	0.84	1.06	0.97
VMH	OTR	Higher in males	0.41	0.12	2.16
Thalamus					
vmThal	V1aR	Higher in females	−0.75	−2.10	−1.02

Only brain regions showing significant main effects of sex or interaction (see Table 1) are included

### Sex differences in V1aR binding density

Sex differences in V1aR binding density were found in 3 of 29 brain regions analyzed (Fig. 2b; see Table 1 for complete statistics). Females had higher V1aR binding density than males in the arcuate nucleus, dorsal lateral septum and ventromedial thalamus (Fig. 6b). While the size of individual sex differences sometimes differed between juveniles and adults (see Table 3 for details), we found no overall difference in the effect sizes of sex differences in V1aR binding between ages (*t*_(4)_ = −2.04; *p* = 0.11).

### Age × sex interaction effects

**Fig. 7:**
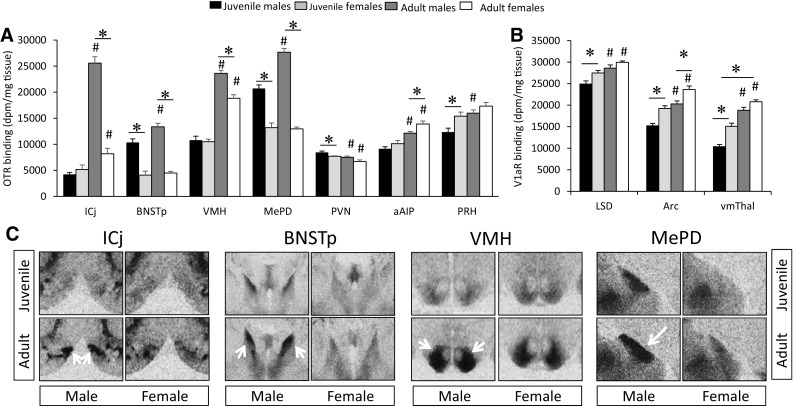
Age and sex differences in OTR (**a**) and V1aR (**b**) binding. OTR binding density in the ICj, BNSTp, VMH, and MePD (**c**) of juvenile and adult male and female rats. OTR binding is higher in adults compared to juveniles in males only in the ICj, BNSTp, and MePD, but in both sexes in the VMH. OTR binding is higher in males than in females at both ages in the BNSTp and MePD, but in adults only in the ICj and VMH. *Bars* indicate mean + SEM; two-way ANOVA (age × sex) with FDR correction for multiple comparisons: *<0.05 versus opposite sex; ^#^<0.05 versus respective juvenile group

Significant age × sex interaction effects were found for OTR binding density in the islands of Calleja, posterior caudate putamen, ventromedial hypothalamus, and posterodorsal medial amygdala (Table 1). Bonferroni post hoc testing revealed that OTR binding density in the islands of Calleja was higher in adults as compared to juveniles in both sexes (males: *p* < 0.001; females: *p* < 0.05) and was higher in adult males as compared to adult females (*p* < 0.001). OTR binding density in the posterior caudate putamen was higher in adult females compared to juvenile females (*p* < 0.01) and compared to adult males (*p* < 0.05). OTR binding density in the ventromedial hypothalamus was higher in adult males compared to juvenile males (*p* < 0.001) and compared to adult females (*p* < 0.001; Fig. 7b). Finally, OTR binding density in the posterodorsal medial amygdala was higher in juvenile males compared to juvenile females (*p* < 0.001) and was higher in adult males compared to juvenile males (*p* < 0.001) and compared to adult females (*p* < 0.001; Fig. 7b). No significant age × sex effects were found for V1aR binding density.

### Similar OTR and V1aR binding density between the ages and sexes

No significant sex or age differences were found in 8 out of 35 brain regions analyzed for OTR binding density: the anterior nucleus accumbens shell, central nucleus accumbens shell, posterior nucleus accumbens shell, dorsal peduncular nucleus, lateral-dorsal BNST, central amygdala, lateral hypothalamus, and posterior caudate putamen (Fig. S1; see Table 1 for statistics) and in 12 out of 29 brain regions analyzed for V1aR binding density: the anterior nucleus accumbens shell, suprachiasmatic nucleus, medial-posterior, lateral-dorsal, and lateral-posterior BNST, lateral periaqueductal gray, lateral hypothalamus, tuberal hypothalamus, anteroventral thalamus, medial thalamus, superficial gray layer of the superior colliculus, and dorsal raphe nucleus (Fig. S1; see Table 1 for statistics).

### Correlational analyses

Correlational analyses were performed to further explore the relationships between OTR and V1aR binding densities across pre-selected brain regions based on the observed age and sex differences in binding densities.

#### Regions with age differences in OTR and V1aR binding: social and spatial memory

**Fig. 8:**
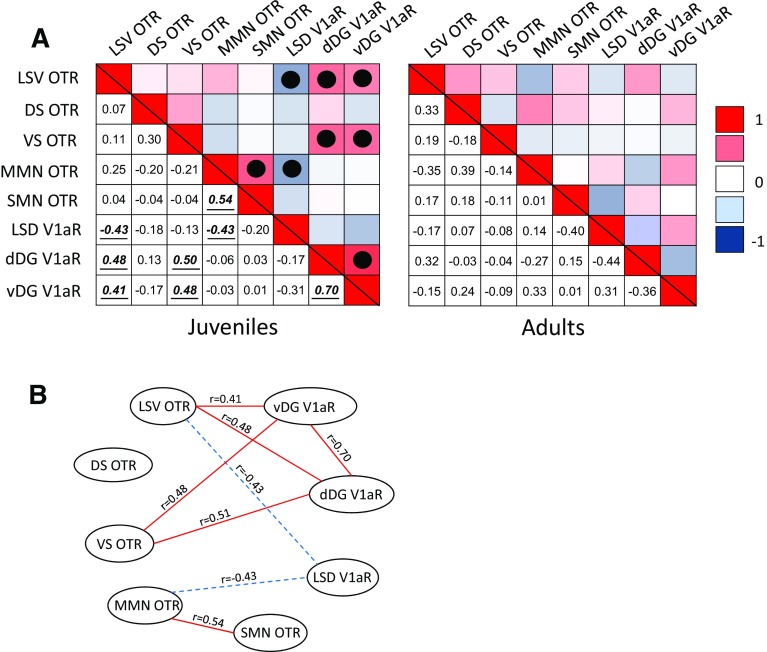
Patterns of covariation between OTR and V1aR binding densities within a network of brain regions involved in social and spatial memory. **a** Heat maps are shown for juveniles (*left*) and for adults (*right*) of both sexes combined. The *upper* and *lower triangle* in each heat map shows the same data. The hue in the *upper triangle* represent the strength of the correlation and *color* indicates the direction of the correlation (*red* = positive; *blue* = negative). The *numbers* in the *lower triangle* represent the correlation coefficient. Significant correlations are marked with a *solid dot* in the *upper triangle* and *bolded*, *italicized*, and *underlined* in the *lower triangle*. Note the presence of eight significant correlations in this network in juveniles and none in adults. **b** Visualization of the network in juveniles showing the correlation coefficients with *solid lines* indicating positive correlations and *dashed lines* indicating negative correlations. Significance set at *p* < 0.05

Age differences in OTR binding densities were found in the ventral lateral septum, dorsal subiculum, ventral subiculum, medial mammillary nucleus and supramammillary nucleus and in V1aR binding densities in the lateral septum and dorsal and ventral dentate gyrus. These brain regions are part of a hypothesized neural network underlying social and spatial memory (Risold and Swanson 1997; Pan and McNaughton 2004; Allen and Hopkins 1989; Meibach and Siegel 1977). Given the importance of social and spatial memory as prerequisites for social decision-making, correlational analyses were run by age to investigate age-specific associations between binding density patterns in these brain regions. Data were collapsed across sexes because no sex differences in binding density were found in these regions. Based on the observed age differences in binding densities in these regions, we hypothesized that OTR and V1aR would show stronger correlations across this neural network in juveniles than in adults. Indeed, eight significant correlations were found across this network in juveniles while none were found in adults (Fig. 8).

#### Regions with both age and sex differences in OTR binding: core nodes of the social behavior network

**Fig. 9:**
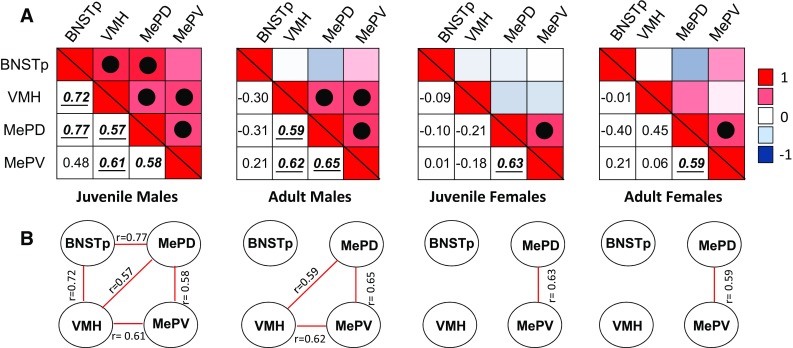
Patterns of covariation between OTR binding densities in the BNSTp, VMH, MePD, and MePV, core brain regions in the social behavior network. Note the strong correlational network in juvenile males and the much weaker correlational network in juvenile and adult females. **a** Heat maps are shown for each age and sex separately with hue representing the strength of the correlation and *color* indicating the direction of the correlation (*red* = positive; *blue* = negative). The *upper* and *lower triangle* in each heat map shows the same data. The hue in the *upper triangle* represent the strength of the correlation and *color* indicates the direction of the correlation (*red* = positive; *blue* = negative). The *numbers* in the *lower triangle* represent the correlation coefficient. Significant correlations are marked with a *solid dot* in the *upper triangle* and *bolded*, *italicized*, and *underlined* in the *lower triangle*. **b** Visualization of the network for each age and sex showing the correlation coefficients with *solid red lines* indicating positive correlations. Significance set at *p* < 0.05

We found both age and sex differences in OTR binding density in the posterior BNST, ventromedial hypothalamus, and posterodorsal and posteroventral medial amygdala (Fig. 7a). These regions are core nodes of the social behavior network (Newman 1999). Because OTR binding density was higher in males and higher in adults in these regions, we hypothesized that OTR binding densities across these regions would correlate more strongly in adults than in juveniles and more strongly in males than in females. Correlational analyses were run separately by age and sex to investigate associations between OTR binding densities in these regions. Out of six possible correlations, five were significant in juvenile males, three were significant in adult males, and one was significant in juvenile and in adult females (Fig. 9).

## Discussion

We hypothesized that age and sex differences in OTR and V1aR binding density would occur in brain regions within the social decision-making network. Moreover, we hypothesized that sex differences in OTR and V1aR binding density would already be present in juveniles. In line with our hypotheses, our analysis revealed a wide array of brain regions, including several within the social decision-making network, in which OTR and V1aR binding densities differ between juvenile and adult rats, as well as between the sexes. More regions displayed denser binding in juveniles than in adults for the OTR, while the opposite was true for the V1aR. Interestingly, sex differences in OTR and V1aR binding densities were less numerous than age differences. The direction of these sex differences was region-specific for the OTR (i.e., OTR binding density was higher in some regions in males and higher in other regions in females), but consistently higher in females for the V1aR. Finally, again in line with our hypothesis, the majority of sex differences in OTR and V1aR binding density were already present in juveniles. Overall, these findings demonstrate that OTR and V1aR binding densities vary greatly between juveniles and adults as well as between the sexes, and highlight the importance of considering developmental stage and sex when making inferences as to the functional roles of OTR and V1aR in the regulation of social behaviors.

Below, we highlight age- and sex-specific patterns of OTR and V1aR binding density with potential relevance to age and sex differences in the regulation of social behavior. Based on previous literature (Young et al. 1999; Knobloch et al. 2012; Caughey et al. 2011; Calcagnoli et al. 2014; Johnson et al. 2016), we make the assumption that higher receptor binding density reflects the likelihood of higher receptor activation. Furthermore, if OTR or V1aR in a given brain region has previously been shown to be involved in the facilitation of a particular behavior, we assume that higher receptor binding density will enhance this facilitation. This assumption also finds support in previous studies (Popik and van Ree 1991; Engelmann and Landgraf 1994; Everts and Koolhaas 1999; Tobin et al. 2010; Veenema et al. 2012; Dumais et al. 2016). However, we realize that these two assumptions might be too simplistic. For example, central OT and AVP system function is intrinsically dependent on OT and AVP release in the brain. Few studies have measured local extracellular OT or AVP release in specific brain regions (Bosch and Neumann 2010; Bosch et al. 2010; Veenema et al. 2010; Lukas et al. 2011) and only one study thus far compared this release between the sexes (Dumais et al. 2016). A proxy measure for OT and AVP release is the analysis of OT and AVP fiber density. Therefore, where possible, we will refer to studies measuring release or fiber density at different ages and in both sexes. Furthermore, OTR and V1aR binding density patterns vary widely across rodent species (Beery et al. 2008; Kelly and Ophir 2015; Albers 2015; Hammock 2015). Therefore, we will focus on discussing relevant research in rats, but we will reference other species where appropriate. With these limitations in mind, the main purpose of this discussion is to provide a conceptual framework in which to further scrutinize the functional roles of age and sex differences in OTR and V1aR binding density.

### Age differences in OTR and V1aR binding density

To the best of our knowledge, only one other study has compared OTR and V1aR binding densities between 35-day-old juvenile rats and adult rats, albeit only in males and in a limited number of brain regions (Lukas et al. 2010). Our results confirm the age differences in OTR and V1aR binding densities reported by Lukas et al. (2010) in the dorsal caudate putamen, ventral lateral septum, and ventromedial hypothalamus for the OTR and dorsal lateral septum, dentate gyrus, and central amygdala for the V1aR. Furthermore, we demonstrate the presence of additional age differences in forebrain and midbrain regions not previously analyzed. Importantly, our design included both males and females, and thus, demonstrates for the first time that age differences in OTR and V1aR binding density are for the most part expressed in both sexes. Although we did not assess vaginal opening or preputial separation in the juvenile rats to determine their reproductive status, these findings demonstrate that OTR and V1aR binding densities at these two life stages are very different and may have important functional consequences for juvenile- versus adult-specific regulation of behavior.

We found that more brain regions displayed higher OTR binding density in juveniles (15 regions) than in adults (10 regions). The age differences we observe in OTR binding densities are largely in line with what is known regarding the developmental trajectory of the OTR in the rat brain (Shapiro and Insel 1989). For example, OTR binding densities were found to be higher in pre-weaning versus adult male rats in the anterior and ventroposterior olfactory nucleus, nucleus accumbens, dorsal caudate putamen, basolateral and basomedial amygdala, dorsal subiculum, and lateral septum (Shapiro and Insel 1989). Our data demonstrate that these age differences in OTR binding density are maintained into the juvenile period. A contrasting pattern of age differences emerged for the V1aR. Here, more brain regions in which V1aR binding differed with age displayed higher binding in adults (10 regions) than in juveniles (7 regions). Previous findings suggest that AVP binding density reaches adult levels in the rat brain by the time of weaning (Tribollet et al. 1991; Snijdewint et al. 1989). Our results challenge that notion by showing that in most brain regions V1aR binding is still less dense in juveniles as compared to adults. Taken together, these results reveal a large network of brain areas in which OTR and V1aR binding densities differ between juveniles and adults of both sexes. The possible functional implications of these age differences are further discussed below.

#### Higher OTR in the juvenile dorsal and ventral striatum may support higher social motivation

Of the regions in which OTR binding was denser in juveniles than in adults, the most robust differences were observed in the anterior nucleus accumbens core and dorsal caudate putamen. These striatal regions have been implicated in the regulation of socially rewarding behaviors (Trezza et al. 2011; Burkett et al. 2011; Resendez et al. 2013), and form key nodes in the social decision-making network (O’Connell and Hoffman 2011, 2012). Moreover, OTR in these regions is involved in mediating socially rewarding behaviors. In detail, activation of the OTR in the striatum promotes partner preference formation and alloparental care in adult female prairie voles (Olazábal and Young 2006; Keebaugh et al. 2015; Ross et al. 2009; Liu and Wang 2003) and conditioned place preference for social stimuli in adult male mice (Dölen et al. 2013). Furthermore, OTR binding is higher in the nucleus accumbens of eusocial naked mole-rats as compared to antisocial cape mole-rats, which may suggest that accumbal OTRs play a role in the non-reproductive social behaviors displayed by the former species (Kalamatianos et al. 2010). However, it should be noted that OTR in the nucleus accumbens is not always associated with more affiliative social behavior repertoires, as evidenced by comparative studies in species such as tuco-tucos (Beery et al. 2008). Thus, cross-species comparisons regarding the potential role of OTR in the striatum should be made cautiously. Importantly, across species, juvenile animals spend more time engaging in peer interactions than do younger or older animals (Doremus-Fitzwater et al. 2010; Blakemore and Mills 2014). These interactions seem to be more rewarding to juveniles, as indicated by the formation of more robust conditioned place preferences for social interaction during the juvenile period as compared to adulthood (Douglas et al. 2004; Trezza et al. 2011; Crone and Dahl 2012). Therefore, we propose that higher OTR binding density in these regions allows for higher OTR activation which may serve to promote enhanced engagement in peer interactions as observed in juveniles, a hypothesis that remains to be tested.

The juvenile period is also characterized by increases in risk-taking, novelty-seeking behavior, and drug abuse (Steinberg et al. 2008), behaviors that have been shown to be mediated by the striatum (Yager et al. 2015; Tops et al. 2014). Additionally, these behaviors are highly sensitive to social context, which can have either a positive or negative influence. For example, the presence of peers can increase alcohol intake and risk-taking behavior in adolescent rats, mice and humans (Varlinskaya et al. 2015; Logue et al. 2014; Chein et al. 2011, Smith et al. 2015), while strong social attachments can reduce the risk of developing a drug addiction in humans (Tops et al. 2014; Young et al. 2014; Buisman-Pijlman et al. 2014; McGregor et al. 2008; Baumgartner et al. 2008). Interestingly, OT has been suggested to play a prominent role in decreasing vulnerability to risk-taking, novelty-seeking, and drug abuse by acting on the striatum to promote social attachments (Tops et al. 2014). It is therefore possible that higher OTR binding density in striatal subregions of juveniles compared to adults provides a mechanism for the enhanced influence of social context on risk-taking and novelty-seeking behaviors in juveniles.

#### Age differences in OTR and V1aR in hippocampus, lateral septum, and mammillary nuclei may underlie age differences in social and spatial memory performance

Age differences in OTR and V1aR binding densities were found in all subregions of the hippocampus, with higher OTR in juveniles in the dorsal and ventral subiculum and higher V1aR in juveniles in the dorsal and ventral dentate gyrus. Age differences were also found in subregions of the lateral septum (ventral lateral septum with higher OTR in juveniles and dorsal lateral septum with lower V1aR in juveniles) and in the mammillary nuclei (medial mammillary and supramammillary nuclei with higher OTR in juveniles). The hippocampus, lateral septum, and mammillary nuclei are highly interconnected brain structures (Risold and Swanson 1997; Pan and McNaughton 2004; Allen and Hopkins 1989; Meibach and Siegel 1977). Furthermore, previous work has shown that both OT and AVP systems regulate social and spatial memory by acting on subregions of the lateral septum and the hippocampal formation. For example, OTR or V1aR blockade in the lateral septum impair social recognition in adult rats (Lukas et al. 2013; Veenema et al. 2012). Moreover, infusion of anti-OT serum into the ventral hippocampus or anti-AVP serum into the dorsal or ventral hippocampus impairs social memory performance in adult male rats (van Wimersma Greidanus and Maigret 1996). Furthermore, AVP injection into the dorsal hippocampus facilitates spatial memory in adult male mice (Paban et al. 2003). Finally, OTR binding densities in the hippocampus, septohippocampal nucleus, and lateral septum correlate with each other and predict socio-spatial memory in adult male prairie voles (Ophir et al. 2012).The mammillary nuclei have also been implicated in spatial memory formation (Méndez-López et al. 2009), although it is currently unknown whether this involves OTR activation. Based on these findings, it is plausible that these brain regions form an interconnected network of structures in which OTR and V1aR activation modulates social and spatial memory performance.

Interestingly, correlational analyses revealed that OTR and V1aR binding densities were more strongly correlated across this interconnected network in juveniles than in adults, with eight significant correlations in juveniles and none in adults. Along with the established roles of OTR and V1aR in these brain regions in adults as discussed above, this finding suggests that age differences in OTR and V1aR binding densities in the hippocampus, lateral septum, and mammillary nuclei may regulate social and spatial memory performance differently in juveniles than in adults. In line with this hypothesis, exposure to a spatial learning task (water maze) evoked neuronal activation in the hippocampus and medial mammillary nucleus in juvenile, but not adult rats (Méndez-López et al. 2009). Furthermore, V1aR blockade in the lateral septum impaired social recognition in adult, but not in juvenile, male and female rats (Veenema et al. 2012). These two studies are the first to suggest age differences in the regulation of social and spatial memory performance by OTR and V1aR in these regions. Further research is needed to provide a causal link between age differences in OTR and V1aR binding density in this network of brain regions and differential regulation of social and spatial memory in juveniles and adults.

#### Higher OTR and V1aR in adulthood in core nodes of the social decision-making network may facilitate adult-specific social behaviors

We found denser OTR binding in adults as compared to juveniles in the ventromedial hypothalamus, posterior BNST, posterodorsal medial amygdala, and medial preoptic area. These brain regions represent core nodes of the social behavior network (Newman 1999) as well as the more expanded, social decision-making network (O’Connell and Hofmann 2011, 2012). Several studies suggest that OTR activation in these regions may enhance the processing of social odor cues. For example, OT facilitates social recognition in adult male and female rats by acting on OTR in the posterior BNST (Dumais et al. 2016) and in adult male rats by acting on OTR in both the medial amygdala (Lukas et al. 2013; Gur et al. 2014) and medial preoptic area (Popik and Van Ree 1991). Additionally, social investigation time correlated positively with OTR binding density in the medial amygdala in female rats (Dumais et al. 2013). These brain regions are also critical to the regulation of reproductive behaviors in both adult males and females (Schulze and Gorzalka 1991; Witt and Insel 1991; McCarthy et al. 1994; Emery and Sachs 1976; Claro et al. 1995; Patil and Brid 2010; Masugi-Tokita et al. 2016; Noack et al. 2015, Kondo 1993; Vochteloo and Koolhaas 1987; Dobolyi et al. 2014) and OTR in each of these brain regions has been implicated in the regulation of adult-specific social behaviors. For example, OT facilitates lordosis responding in adult female rats by acting on OTR in the ventromedial hypothalamus (McCarthy et al. 1994; Schulze and Gorzalka 1991) and facilitates maternal aggression in lactating rats by acting on OTR in the posterior BNST and medial preoptic area (Consiglio et al. 2005; Pedersen et al. 1994). Furthermore, in adult male rats, duration of aggressive behavior correlated positively with OTR binding density in the posterior BNST (Calcagnoli et al. 2014). To the best of our knowledge, the role of OTR in these brain regions is unknown in juveniles. Therefore, it would be of interest to determine whether higher OTR binding density in these brain regions in adults as compared to juveniles serves to enhance the processing of social odor cues and promote adult reproductive behaviors.

We also found higher V1aR binding density in the ventral pallidum of adults as compared to juveniles. The ventral pallidum is part of the mesolimbic reward system and forms a node in the social decision-making network (O’Connell and Hoffman 2011, 2012). V1aR in the ventral pallidum has been associated with mating-induced social affiliation in adult voles. Here, V1aR binding is denser in monogamous male prairie voles than in polygynous male montane voles (Young et al. 2001). Furthermore, V1aR activation in the ventral pallidum facilitates partner preference formation in monogamous male prairie voles (Lim and Young 2004; Pitkow et al. 2001) and experimentally increased V1aR in the ventral pallidum induces monogamous-like partner preference formation in polygynous male meadow voles (Lim et al. 2004). Increased V1aR expression in the ventral pallidum of male prairie voles enhanced mating-induced neuronal activation, as evidenced by Fos induction (Lim and Young 2004). Interestingly, paired female prairie voles show higher V1aR binding density in the ventral pallidum than do their single counterparts (Zheng et al. 2013), suggesting that ventral pallidum V1aR may also play a role in the regulation of mating-induced partner preference formation in females. Moreover, ventral pallidum V1aR binding density is higher in the early stages of pregnancy as compared to both the later stages of pregnancy and nonpregnancy (Ophir et al. 2013), which may also reflect mating-induced changes in the V1aR. Ophir et al. (2013) has suggested that high ventral pallidum V1aR binding density may facilitate social bonding and that low ventral pallidum V1aR binding density may accommodate social promiscuity (Ophir et al. 2013). Although rats are not known as a monogamous species, under certain conditions male rats do form mating preferences for a familiar female rat (Ismail et al. 2009). Taken together, it is plausible that the V1aR in the ventral pallidum of adult rats facilitates preferences for the opposite sex and/or mating behaviors. This hypothesis has yet to be explored in rats, and thus offers an interesting avenue for future research.

#### Higher OTR and V1aR in cortical areas in adulthood may facilitate social behavior by improving signal-to-noise ratio, and therefore, enhancing the salience of social cues

Adult rats showed higher OTR and V1aR binding density than juvenile rats in seven out of eight cortical regions analyzed. These were the infralimbic, prelimbic, anterior insular, medial insular, and perirhinal cortices for the OTR and the piriform and sensory (S1) cortices for V1aR. Infralimbic and prelimbic OTR binding was very low as compared to OTR binding in any of the other regions analyzed. Nevertheless, recent studies indicate that OTR in these areas are functional and play a role in reproduction-related social behaviors. For example, impairing OTR function in the medial prefrontal cortex (including the infralimbic and prelimbic cortices; either by pharmacological inhibition of the OTR, deletion of the OTR gene, or chronic silencing of OTR expressed on interneurons) reduced social investigation of adult male mice by adult female mice, especially when the subject females were in estrus (Nakajima et al. 2014). Additionally, OTR blockade in the prelimbic cortex impaired maternal care behavior in lactating female rats (Sabihi et al. 2014). Little is known about the mechanisms and neural circuits by which activation of OTR modulates these behaviors. However, a recent study focusing on the auditory cortex may shed light on a potential mechanism of action. Here, OT facilitated pup retrieval in adult virgin female mice by balancing excitatory and inhibitory signals in the auditory cortex, thereby improving the signal-to-noise ratio (Marlin et al. 2015). Improved excitatory/inhibitory balance is a feature naturally observed in the auditory cortex of lactating female mice (Cohen et al. 2011; Liu et al. 2006; Rothschild et al. 2013). Marlin et al. (2015) speculate that this reshaping of neuronal responses by OT may enhance the salience of pup distress calls and allow for the appropriate behavioral response, i.e., pup retrieval. Interestingly, the signal-to-noise ratio of cortical electrical signals is lower in adolescents as compared to adults in rats and humans (Segalowitz and Davies 2004; Sturman and Moghaddam 2011). Moreover, OT acting on OTR was shown to balance excitatory and inhibitory input in the hippocampus in juvenile rats (Owen et al. 2013). These findings may suggest that cortical OTR play a role in mediating an optimal excitatory/inhibitory balance irrespective of age, while our current findings may suggest that this function is heightened in adults compared to juveniles because of higher OTR, and perhaps V1aR, binding density in cortical regions.

#### Sex differences in OTR and V1aR binding densities

We are the first to directly compare OTR and V1aR binding densities in males and females and juveniles and adults in one design. Although less numerous than age differences, we found a number of sex differences in OTR and V1aR binding density. Importantly, we are the first to demonstrate that in most brain regions, sex differences in OTR and V1aR binding density are present at postnatal day 35, and that sex differences in OTR and V1aR occur in brain areas in which binding density is higher in adults than in juveniles. It should be noted that we did not control for possible effects of estrous cycle phase on OTR and V1aR binding density. However, previous findings in adult rats suggest that estrous phase has limited impact on sex differences in OTR and V1aR binding density (Dumais et al. 2013; Dumais and Veenema 2016a, b). Moreover, OTR and V1aR binding density variability (as interpreted by the average standard deviation of binding density in all brain regions) was no greater in females than in males, suggesting that it is unlikely that estrous phase had a large impact on the observed sex differences in OTR and V1aR binding density.

OTR binding was denser in males than in females in the posterior BNST, ventromedial hypothalamus, medial preoptic area and posterodorsal and posteroventral medial amygdala. These sex differences confirm those of previous studies in rats (Uhl-Bronner et al. 2005; Dumais et al. 2013), suggesting that these are highly robust and persistent sex differences. On the other hand, we also found sex differences in OTR and V1aR binding density (such as in the islands of Calleja, intermediate lateral septum, anterior insular cortex, and perirhinal cortex for the OTR and in the dorsal lateral septum, arcuate nucleus, and ventromedial thalamus for the V1aR) that were either not found or not characterized in previous studies of receptor binding (Dumais et al. 2013; Dumais and Veenema 2016a, b) or mRNA (Szot et al. 1994). Importantly, one notable difference between our current study and previous work (Dumais et al. 2013; Dumais and Veenema 2016a) is the behavioral experience of the subjects. In this study, rats were socially housed but underwent no behavioral testing prior to brain tissue collection for receptor binding. In the previous studies, rats were singly housed and exposed to several social behavior tests 2 weeks prior to brain tissue collection (Dumais et al. 2013; Dumais and Veenema 2016a). Both OTR and V1aR binding densities can be altered by life experiences, such as parenthood and early life stress (Lukas et al. 2010; Bales and Perkeybile 2012; Bosch et al. 2010; Bosch and Neumann 2008; Lukas et al. 2010; Dumais and Veenema 2016a), and this may occur in sex-specific ways (Curley et al. 2009). Given this plasticity, it is likely that differences in social experiences contribute to the inconsistencies in sex differences in OTR and V1aR binding densities between these studies. Notably, those sex differences in OTR binding density that were replicated were of higher magnitude, making it possible that if binding density were to be changed due to experience that this may not have obscured the sex difference in these regions. Conversely, experience-induced plasticity may be able to change the presence or direction of sex differences in OTR and V1aR binding density in those brain regions showing smaller magnitude sex differences.

#### Sex differences in correlations between OTR binding densities in the posterior BNST, ventromedial hypothalamus, and medial amygdala: implications for the regulation of sex differences in behavior

OTR binding densities across the posterior BNST, ventromedial hypothalamus, posterodorsal medial amygdala, and posteroventral medial amygdala correlated more strongly in juvenile and adult males than in females. It has been suggested that the relative activation of these regions, along with other regions within the social behavior network, determine the type of social behavior expressed (Newman 1999). Accordingly, OTR in the posterior BNST, ventromedial hypothalamus, and medial amygdala may play a key role in this relative activation. It would be interesting to test whether higher and more strongly correlated OTR binding densities in these regions in males than in females provides a mechanism to mediate sex-specific regulation of particular social behaviors in both juveniles and adults.

#### Most sex differences in OTR and V1aR binding density are already present at juvenile age

A novel and important finding of this study is the early emergence of sex differences in OTR and V1aR binding density. With the notable exceptions of the ventromedial hypothalamus and anterior insula, all sex differences found were already present at the juvenile age. The function of the early presence of these sex differences is unclear, but may suggest a role for OTR and V1aR in the sex-specific regulation of juvenile, in addition to adult, social behaviors. Interestingly, OTR and V1aR were found to be involved in the sex-specific regulation of juvenile social play behavior (Veenema et al. 2013; Bredewold et al. 2014). Further research in juveniles is required to understand, more completely, the sex-specific roles of OT and AVP systems in the regulation of social behavior. Moreover, further research is also needed to clarify the mechanisms by which sex differences in OTR and V1aR binding density in the brain are created.

#### Sex differences in OTR and V1aR binding density occur in brain regions with denser binding in adults than in juveniles: 1. Possible role in both age- and sex-specific regulation of social behavior

We found that in most of the brain regions in which OTR and V1aR binding density differs between the sexes, binding is denser in adult as compared to juvenile rats. These brain regions are the islands of Calleja, ventromedial hypothalamus, posterior BNST, posterodorsal medial amygdala, anterior insular and perirhinal cortex for the OTR and the dorsal lateral septum, arcuate nucleus, and ventromedial thalamus for the V1aR. These may be regions in which OTR and V1aR play a role in both age and sex differences in the regulation of social behavior. For example, the AVP system in the lateral septum is involved in both age- and sex-specific regulation of social recognition in rats (Veenema et al. 2012). Interestingly, the age difference in V1aR binding density in the lateral septum corresponds with a similar age difference in AVP fiber density in the lateral septum (De Vries et al. 1981), further supporting an age-specific role of the AVP system in the lateral septum. However, the sex difference in V1aR binding density (higher in females) is opposite to the sex difference in AVP fiber density (higher in males) in the lateral septum (De Vries et al. 1981), making it more difficult to understand the behavioral consequences in either sex. Furthermore, we recently showed that the OTR in the posterior BNST plays a role in the sex-specific regulation of social recognition of adult rats, with higher OTR binding density associated with higher OT release in males than in females (Dumais et al. 2016). These studies highlight the importance of determining whether age and sex differences in OTR and V1aR binding density are accompanied by age and sex differences in OT and AVP innervation/release in the same regions. This knowledge may help to understand the functional consequences of age and sex differences in OTR and V1aR binding density.

#### Sex differences in OTR and V1aR binding density occur in brain regions with denser binding in adults than in juveniles: 2. Possible role for circulating gonadal hormones after puberty

The age-dependent increase in OTR and V1aR binding density predominantly occurs in regions that also show sex differences in receptor binding density. It is therefore likely that these regions are sensitive to gonadal steroids and that the age-dependent increases are induced by increases in gonadal hormone levels at the onset of puberty and thereafter. Indeed, evidence suggests that the expression of OTR, and to some extent V1aR, requires circulating gonadal hormones. For example, adult gonadectomy reduced the amount of [^3^H] OT binding in the islands of Calleja and ventromedial hypothalamus in both male and female rats (Tribollet et al. 1990). Furthermore, testosterone or estradiol replacement after gonadectomy restored [^3^H] OT binding in the islands of Calleja and ventromedial hypothalamus to pre-gonadectomy levels in female rats (Tribollet et al. 1990). Moreover, adult treatment with an aromatase inhibitor (which prevents the conversion of testosterone to estradiol) decreased [^3^H] OT binding in the ventromedial hypothalamus, albeit not as robustly as gonadectomy, in male rats (Tribollet et al. 1990). It should be noted that the above discussed findings are based on the use of a nonselective OT-radiolabeled ligand and a relatively low numbers of animals (3–4) per group (Tribollet et al. 1990). These factors may also explain why adult gonadectomy was not found to alter [^3^H] AVP binding in rats (Tribollet et al. 1990). In contrast, using a more specific [^125^I] V1aR-radiolabeled ligand, adult gonadectomy decreased V1aR binding density in the BNST and ventromedial hypothalamus, among other regions, in male hamsters (Delville and Ferris 1995; Johnson et al. 1995; Young et al. 2000). These results lend support to the idea that gonadal hormones are important for the maintenance of OTR binding density. However, comparative studies will be required to demonstrate that gonadal hormone removal in adulthood reduces OTR or V1aR binding density to juvenile levels.

#### Regions of functional significance, despite a lack of age and sex differences in OTR and V1aR binding density

It is important to note that many brain regions show dense OTR and V1aR binding without age and sex differences. For the OTR, these areas include the dorsal peduncular nucleus, anterior, central and posterior nucleus accumbens shell, posterior caudate putamen, laterodorsal BNST, central amygdala and lateral hypothalamus. For the V1aR these areas include all subdivisions of the BNST, the anterior nucleus accumbens shell, the lateral and tuberal hypothalamus, the suprachiasmatic nucleus, the anteroventral and medial thalamus, the lateral periaqueductal gray, the superficial gray layer of the superior colliculus, and the dorsal raphe nucleus. The latter two represent regions in which, to the best of our knowledge, V1aR binding has not been analyzed before. Interestingly, V1aR binding has been observed in the periaqueductal gray of *Scotinomys teguina*, a species of singing mouse, where it may play a role in the generation of vocalizations (Campbell et al. 2009). OTR and V1aR in these brain regions likely serve important functions that may not differ across development or between the sexes. Yet the absence of an age or sex difference in OTR and V1aR binding density does not exclude the possibility of age- or sex-specific involvement of OTR and V1aR in the regulation of social behavior. It would therefore be of interest to further explore the functions of OTR and V1aR in these brain regions at both juvenile and adult ages and in both males and females.

#### Opposing roles of OTR and V1aR in the central amygdala: possible role in age-specific anxiety or fear responding

OT in the central amygdala has been shown to decrease fear responding in adult male and female rats (Viviani et al. 2011; Knobloch et al. 2012). This is thought to be mediated through local network activity involving both OTR and V1aR. In detail, activation of OTR-expressing central amygdala neurons inhibits the activation of V1aR-expressing central amygdala neurons (Huber et al. 2005). This pathway may allow for OTR and V1aR within the central amygdala to have opposing responses on anxiety and fear. Interestingly, OTR binding density in the central amygdala doesn’t show age or sex differences, but V1aR binding density in the central amygdala is higher in juveniles than in adults. This may result in more V1aR relative to OTR activation in juveniles as compared to adults. Although the role of V1aR and OTR in the central amygdala in juvenile rats in unknown, this could indicate that the V1aR plays a larger functional role than the OTR in juveniles, and may result in age-specific anxiety/fear responses. This remains to be tested.

## Conclusion

In conclusion, our work demonstrates the presence of age and sex differences in OTR and V1aR binding densities in the rat brain, particularly, in brain regions that form the social decision-making network (O’Connell and Hoffman 2011, 2012). We discussed that age differences in OTR and V1aR binding densities may have implications for the regulation of social motivation (through higher OTR binding in striatal areas) and social and spatial memory (through age differences in OTR and V1aR in the hippocampus, lateral septum and mammillary nuclei) in juveniles, and for the regulation of adult-typical social behaviors (through higher OTR and V1aR in nodes of the social decision-making network and higher OTR in cortical areas). We further showed, for the first time, that most sex differences in OTR and V1aR binding are already present at juvenile age and are found in regions showing a further increase in binding density in adults. We discussed that these regions represent a unique network in which to expect both age and sex differences in the regulation of social behavior. Further research is needed to determine (a) the behavioral relevance of age and sex differences in OTR and V1aR, and (b) the neural and molecular mechanisms that underlie these age and sex differences.

## Electronic supplementary material

Below is the link to the electronic supplementary material.

Supplementary Fig. 1. Brain regions in which no age or sex differences in OTR (A) or V1aR (B) binding densities in the rat brain were found. OTR binding was analyzed on three-day exposure films for the DP, BNSTdl and CeA and on nine-day exposure films for all other regions. V1aR binding was analyzed on four-day exposure films. Bars indicate mean + SEM; two-way ANOVA (age x sex) with FDR α < 0.020 for OTR binding and FDR α < 0.015 for V1aR binding. (PPTX 77 kb)

## Abbreviations

aAcbC: Anterior nucleus accumbens core
aAIP: Anterior agranular insular cortex
aAcbSh: Anterior nucleus accumbens shell
cAcbSh: Central nucleus accumbens shell
pAcbSh: Posterior nucleus accumbens shell
AODL: Anterior olfactory nucleus, dorsolateral part
AOM: Anterior olfactory nucleus, medial part
AOVP: Anterior olfactory nucleus, ventroposterior part
Arc: Arcuate nucleus
AVP: Arginine vasopressin
avThal: Anteroventral thalamic nucleus
BLA: Basolateral amygdala
BMA: Basomedial amygdala
BNSTld: Bed nucleus of the stria terminalis, lateral division, dorsal part
BNSTlp: Bed nucleus of the stria terminalis, lateral division, posterior part
BNSTmp: Bed nucleus of the stria terminalis, posteromedial part
BNSTp: Bed nucleus of the stria terminalis, posterior part
CeA: Central amygdala
Cl: Claustrum
dCPu: Dorsal caudate putamen
DP: Dorsal peduncular cortex
DRN: Dorsal raphe nucleus
DS: Dorsal subiculum
GrDG: Granular layer of the dentate gyrus
ICj: Islands of Calleja
IL: Infralimbic cortex
IPAC: Interstitial nucleus of the posterior limb of the anterior commissure
LH: Lateral hypothalamus
LPAG: Lateral periaqueductal gray
LSD: Lateral septum, dorsal part
LSI: Lateral septum nucleus, intermediate part
LSV: Lateral septum nucleus, ventral part
MA3: Medial accessory oculomotor nucleus
mAIP: Medial agranular insular cortex
mCPu: Medial caudate putamen
MePD: Medial amygdala, posterodorsal part
MePV: Medial amygdala, posteroventral part
MMN: Medial mammillary nucleus
moDG: Molecular layer of the dentate gyrus
MPOA: Medial preoptic area
mTHal: Medial thalamic nucleus
Nv: Navicular nucleus of the basal forebrain
OT: Oxytocin
OTR: Oxytocin receptor
pAcbSh: Nucleus accumbens shell posterior part
pCPu: Posterior caudate putamen
Pir: Piriform cortex
PRH: Perirhinal cortex
PrL: Prelimbic cortex
PVN: Paraventricular hypothalamic nucleus
PVT: Paraventricular thalamic nucleus
S1: Primary somatosensory cortex
SCN: Suprachiasmatic nucleus
SMN: Supramammillary nucleus
SPFPC: Subparafascicular nucleus
stg: Stigmoid hypothalamic nucleus
Sug: Superficial gray layer of the superior colliculus
tuLH: Tuberal region of the lateral hypothalamus
V1aR: Vasopressin V1a receptor
VMH: Ventromedial hypothalamic nucleus
vmThal: Ventromedial thalamic nucleus
VP: Ventral pallidum
VS: Ventral subiculum
