# Supplementary material for: Age and sex differences in oxytocin and vasopressin V1a receptor binding densities in the rat brain: focus on the social decision-making network
Source: Brain Struct Funct. 2016 Jul 7;222(2):981–1006. doi: 10.1007/s00429-016-1260-7 (PMC5334374; doi:10.1007/s00429-016-1260-7)
Supplement: Supplementary file 1 — Supplementary Fig. 1. Brain regions in which no age or sex differences in OTR (A) or V1aR (B) binding densities in the rat brain were found. OTR binding was analyzed on three-day exposure films for the DP, BNSTdl and CeA and on nine-day exposure films for all other regions. V1aR binding was analyzed on four-day exposure films. Bars indicate mean + SEM; two-way ANOVA (age x sex) with FDR α < 0.020 for OTR binding and FDR α < 0.015 for V1aR binding. (PPTX 77 kb) [file 429_2016_1260_MOESM1_ESM.pptx]

## Slide 1
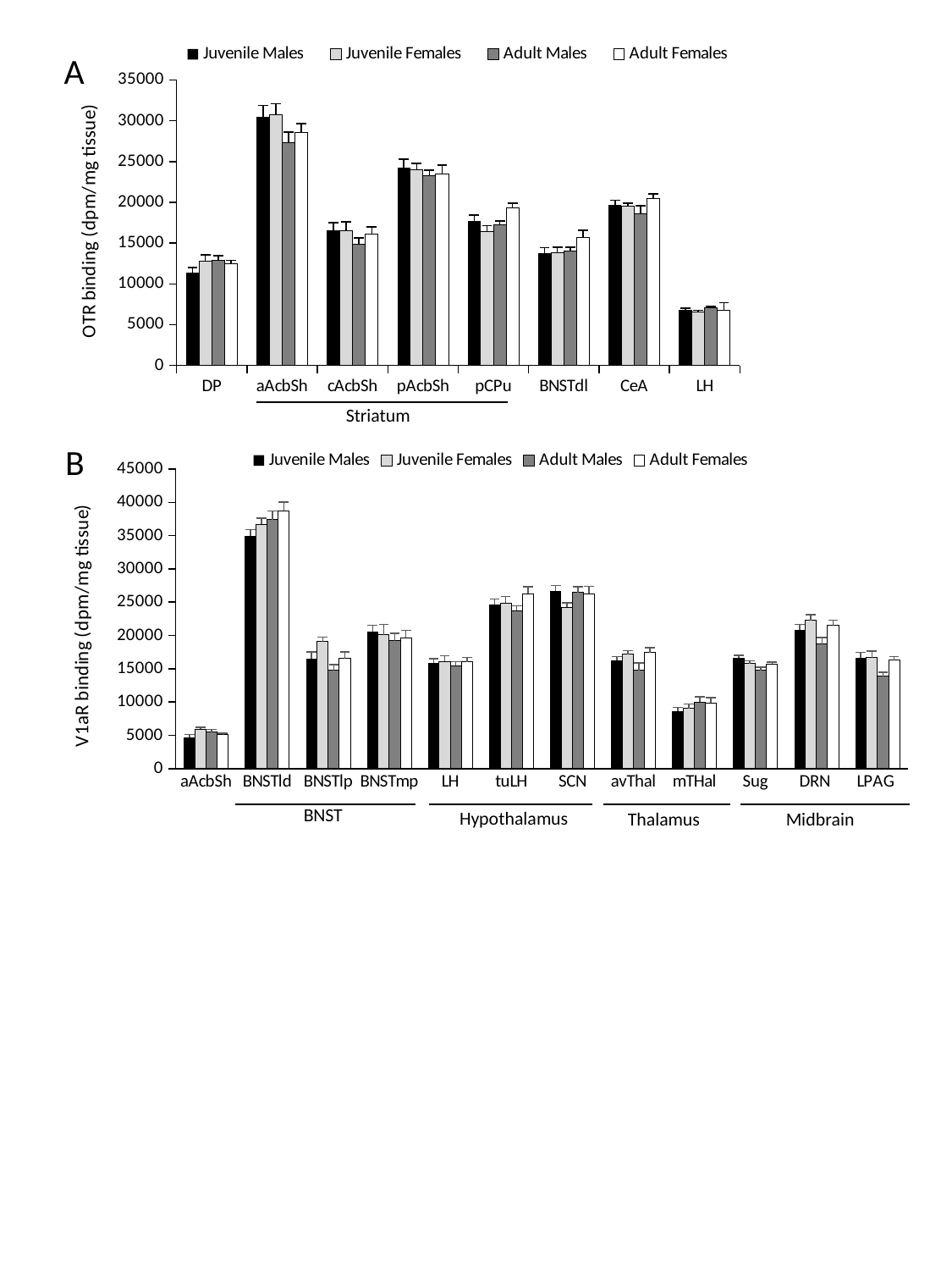

### Chart
| Category | Juvenile Males | Juvenile Females | Adult Males | Adult Females |
|---|---|---|---|---|
| DP | 11400.52655569231 | 12823.148809615386 | 12920.425925833333 | 12457.448975000001 |
| aAcbSh | 30449.605 | 30717.2336 | 27322.723 | 28532.0904 |
| cAcbSh | 16540.75376984127 | 16516.78754578755 | 14896.204365079364 | 16122.138492063494 |
| pAcbSh | 24237.9115384615 | 24042.97692307692 | 23222.00694444445 | 23431.68333333333 |
| pCPu | 17706.106684615384 | 16381.141025384613 | 17286.714584166664 | 19286.42361166667 |
| BNSTdl | 13726.929486923076 | 13844.347221666669 | 14063.215277499998 | 15697.076389166668 |
| CeA | 19644.615384615383 | 19523.615384615383 | 18606.583333333336 | 20495.0 |
| LH | 6787.662782461538 | 6577.536975000001 | 7023.639127899999 | 6807.3583194444445 |A
Striatum
B
### Chart
| Category | Juvenile Males | Juvenile Females | Adult Males | Adult Females |
|---|---|---|---|---|
| aAcbSh | 4746.6755 | 5900.9068 | 5508.999 | 5110.018 |
| BNSTld | 34911.959180000005 | 36716.84213916668 | 37375.205082500004 | 38735.07399899999 |
| BNSTlp | 16525.194781538466 | 19094.973695 | 14738.455334166667 | 16560.915649 |
| BNSTmp | 20615.653846153848 | 20091.73076923077 | 19202.409090909092 | 19674.4 |
| LH | 15907.191779166666 | 16117.330076923077 | 15489.737774999998 | 16039.75641111111 |
| tuLH | 24681.03647916667 | 24900.93116 | 23740.389754166667 | 26256.39285 |
| SCN | 26686.18877923077 | 24266.608568461543 | 26449.002617500002 | 26266.879708 |
| avThal | 16289.0013 | 17170.5926 | 14768.3128 | 17462.1604 |
| mTHal | 8636.6824 | 9074.13 | 9977.4562 | 9891.8242 |
| Sug | 16599.062853076925 | 15780.842993846154 | 14775.078151666667 | 15696.096967999998 |
| DRN | 20816.33855666667 | 22279.816049999998 | 18728.145924545457 | 21494.696517 |
| LPAG | 16583.358516153847 | 16664.866758461536 | 13844.468749999998 | 16259.012499999999 |BNST
Hypothalamus
Thalamus
Midbrain
